# GATA6 regulates EMT and tumour dissemination, and is a marker of response to adjuvant chemotherapy in pancreatic cancer

**DOI:** 10.1136/gutjnl-2015-311256

**Published:** 2016-06-20

**Authors:** Paola Martinelli, Enrique Carrillo-de Santa Pau, Trevor Cox, Bruno Sainz, Nelson Dusetti, William Greenhalf, Lorenzo Rinaldi, Eithne Costello, Paula Ghaneh, Núria Malats, Markus Büchler, Marina Pajic, Andrew V Biankin, Juan Iovanna, John Neoptolemos, Francisco X Real

**Affiliations:** 1 Epithelial Carcinogenesis Group, Spanish National Cancer Research Center-CNIO, Madrid, Spain; 2 Cancer Progression and Metastasis Group, Institute for Cancer Research, Medical University Wien, Vienna, Austria; 3 Cancer Research UK Liverpool Clinical Trials Unit, University of Liverpool, Liverpool, UK; 4 NIHR Liverpool Pancreas Biomedical Research Unit, Department of Molecular and Clinical Cancer Medicine, University of Liverpool, Liverpool, UK; 5 Department of Preventive Medicine, Public Health and Microbiology, Universidad Autónoma de Madrid, Madrid, Spain; 6 Centre de Recherche en Cancérologie de Marseille (CRCM), INSERM U1068, CNRS UMR 7258, Aix-Marseille Université and Institut Paoli-Calmettes, Parc Scientifique et Technologique de Luminy, Marseille, France; 7 Institute for Research in Biomedicine (IRB), Barcelona, Spain; 8 Genetic and Molecular Epidemiology Group, Spanish National Cancer Research Center-CNIO, Madrid, Spain; 9 Department for General, Visceral and Transplantation Surgery, University Hospital Heidelberg, Heidelberg, Germany; 10 Cancer Division, The Kinghorn Cancer Centre, Garvan Institute of Medical Research, Sydney, Australia; 11 Wolfson Wohl Cancer Research Centre, Institute of Cancer Sciences, University of Glasgow, Glasgow, UK; 12 West of Scotland Pancreatic Unit, Glasgow Royal Infirmary, Glasgow, UK; 13 South Western Sydney Clinical School, Faculty of Medicine, University of NSW, Liverpool, Australia; 14 Departament de Ciències Experimentals i de la Salut, Universitat Pompeu Fabra, Barcelona, Spain

**Keywords:** CANCER GENETICS, EPITHELIAL DIFFERENTIATION, GENE REGULATION, PANCREATIC CANCER, ADJUVANT TREATMENT

## Abstract

**Background and aims:**

The role of GATA factors in cancer has gained increasing attention recently, but the function of GATA6 in pancreatic ductal adenocarcinoma (PDAC) is controversial. GATA6 is amplified in a subset of tumours and was proposed to be oncogenic, but high GATA6 levels are found in well-differentiated tumours and are associated with better patient outcome. By contrast, a tumour-suppressive function of GATA6 was demonstrated using genetic mouse models. We aimed at clarifying GATA6 function in PDAC.

**Design:**

We combined GATA6 silencing and overexpression in PDAC cell lines with GATA6 ChIP-Seq and RNA-Seq data, in order to understand the mechanism of GATA6 functions. We then confirmed some of our observations in primary patient samples, some of which were included in the ESPAC-3 randomised clinical trial for adjuvant therapy.

**Results:**

GATA6 inhibits the epithelial–mesenchymal transition (EMT) in vitro and cell dissemination in vivo. GATA6 has a unique proepithelial and antimesenchymal function, and its transcriptional regulation is direct and implies, indirectly, the regulation of other transcription factors involved in EMT. GATA6 is lost in tumours, in association with altered differentiation and the acquisition of a basal-like molecular phenotype, consistent with an epithelial-to-epithelial (ET^2^) transition. Patients with basal-like GATA6^low^ tumours have a shorter survival and have a distinctly poor response to adjuvant 5-fluorouracil (5-FU)/leucovorin. However, modulation of GATA6 expression in cultured cells does not directly regulate response to 5-FU.

**Conclusions:**

We provide mechanistic insight into GATA6 tumour-suppressive function, its role as a regulator of canonical epithelial differentiation, and propose that loss of GATA6 expression is both prognostic and predictive of response to adjuvant therapy.

**Significance of this study**

**What is already known on this subject?**

- GATA6 maintains the epithelial differentiation in the mouse pancreas and suppresses mutant KRas-driven tumourigenesis in the mouse.
- Pancreatic tumours of the classical subtype, characterised by better outcome, have high GATA6 expression.
- *GATA6* is amplified in a subset of pancreatic tumours, and its overexpression increases proliferation of pancreatic cancer cells in vitro.
- Patients with tumours carrying *GATA6* amplifications/copy number gains survive longer.

**What are the new findings?**

- GATA6 regulates epithelial–mesenchymal transition (EMT) in pancreatic cancer cells through a unique mechanism, both direct and indirect, controlling both the epithelial and the mesenchymal transcriptional programmes.
- GATA6 suppresses the ectopic expression of a basal-like molecular phenotype, similar to the one described in breast and bladder cancer, which is activated in a subset of GATA6^low^tumours.
- Patients with basal-like GATA6^low^ tumours show a worse survival than those with GATA6^medium^ or GATA6^high^ tumours.
- Patients with GATA6^low^ tumours have a worse outcome when treated with 5-fluorouracil (5-FU)/leucovorin adjuvant therapy, compared with patients with GATA6^high^ tumours, while treatment with gemcitabine has the same effect on both groups

**How might it impact on clinical practice in the foreseeable future?**

- We finally provide an explanation to the conundrum derived from the observation that GATA6 is amplified in a subset of tumours; yet, patients with high GATA6 survive longer.
- GATA6 expression could be a marker for patients’ prognosis.
- If confirmed in an independent study, our observation that patients with GATA6^low^ tumours have a worse outcome when treated with 5-FU/leucovorin adjuvant therapy could guide the choice of treatment for patients with pancreatic cancer.

## Introduction

Pancreatic ductal adenocarcinoma (PDAC), the most common type of pancreatic cancer, has a dismal prognosis^1^ ^2^ with a 5-year survival of 25%–30% after resection and adjuvant chemotherapy with either gemcitabine or 5-fluorouracil (5-FU)+leucovorin or gemcitabine.^3–7^ Most patients present with advanced disease and are not eligible for surgery. Gemcitabine is the mainstay of therapy for locally advanced and metastatic disease. Recently, gemcitabine+nab-paclitaxel and FOLFIRINOX combination chemotherapies showed a modest improvement in survival of patients with advanced disease.^8^ ^9^

Exome/genome sequencing of PDAC has revealed a complex pattern of genetic alterations, affecting multiple core signalling pathways.^10^ The few frequently altered genes (*KRAS*, *CDKN2A*, *TP53*, *SMAD4*) have proven difficult to target therapeutically. The remaining alterations occur in <10% of tumours and, therefore, are not ideal targets for new therapies. Patient stratification for treatment selection is unfeasible because of the scarcity of pathological/molecular markers that can reliably predict therapeutic response. The recent report of high hENT-1 tumour protein levels being associated with response to gemcitabine is promising, but needs to be replicated in prospective studies.^11^ The identification of new therapeutic targets and markers for patient stratification and targeted treatment are the two priorities.

Omics technologies provide a new molecular taxonomy of cancer. In PDAC, few studies have aimed at a molecular-based classification. Collisson *et al*^12^ identified three PDAC subtypes: classical, exocrine and mesenchymal-like. Classical tumours showed high GATA6 mRNA expression, and patients had a significantly better outcome. Cells with a classical phenotype showed distinct response to chemotherapy in vitro*.*^12^ GATA6 belongs to a family of transcription factors that bind to the (A/T)GATA(A/G) consensus sequence to activate or repress gene expression.^13^ GATA factors are important for cell differentiation, and *GATA6* is essential for the maintenance of the exocrine pancreas in adult mice.^14^ An oncogenic role was proposed for GATA6 in PDAC based on the occurrence of *GATA6* gains/amplifications in a small proportion of tumours.^15^ ^16^ However, high *GATA6* copy number is significantly associated with a better outcome in patients with PDAC,^17^ suggesting that its function could be more complex than originally proposed. A tumour-suppressive role of GATA6 has been recently postulated in PDAC mouse models,^18^ ^19^ where it regulates differentiation-related as well as cancer-related transcriptional programmes.

Here, we show that, in human PDAC cells, GATA6 inhibits de-differentiation and epithelial–mesenchymal transition (EMT), both directly and indirectly, through a unique mechanism that involves the regulation of transcription factors, including FOXA1/2. Consistently, loss of GATA6 in PDAC primary samples is associated with altered differentiation and shorter overall patient survival. Finally, the analysis of tumour samples from the ESPAC-3 randomised adjuvant chemotherapy trial^7^ shows that low GATA6 expression can predict worse response to adjuvant 5-FU/leucovorin.

## Results

### *GATA6* maintains the canonical epithelial phenotype in PDAC cells

**Figure 1:**
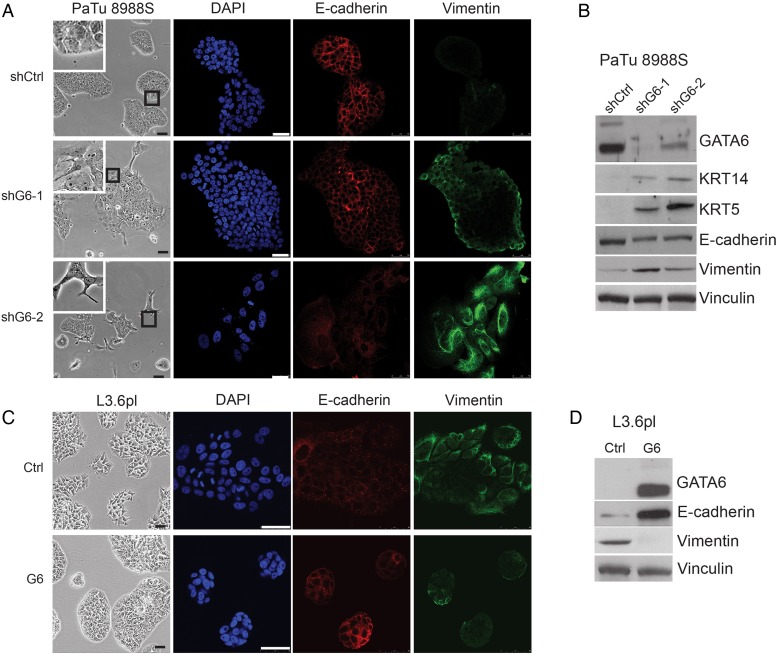
GATA6 is required for the maintenance of the epithelial phenotype of pancreatic ductal adenocarcinoma (PDAC) cells. (A) Top: phase contrast microphotographs of PaTu8988S cells infected with either shCtrl or two different GATA6-targeting shRNAs (shG6-1 and shG6-2). Higher magnification of the highlighted region is shown in the inset. Bottom: expression of E-cadherin and vimentin detected by immunofluorescence. Nuclear counterstaining with diamidino-2-phenylindole (DAPI) is shown separately. Scale bars: 50 μm. (B) Expression of GATA6, KRT5, KRT14, E-cadherin and vimentin, detected by western blotting, in total lysates from PaTu8988S cells infected with the indicated constructs. Vinculin was used as a loading control. (C) Left: L3.6pl cells infected with either an empty vector (Ctrl) or a GATA6-overexpressing vector (G6). Right: expression of E-cadherin, and vimentin detected by immunofluorescence. Nuclear counterstain with DAPI is shown separately. Scale bars: 50 μm. (D) Expression of GATA6, E-cadherin and vimentin detected by western blotting in total lysates from L3.6pl cells infected with the indicated constructs. Vinculin was used as a loading control.

To determine the function of GATA6, we analysed its expression in a panel of PDAC cell lines to select the optimal models for loss-of-function and gain-of-function analyses (see online supplementary figure S1). We silenced GATA6 in three PDAC cell lines, including one with *GATA6* amplification (A13B),^16^ using two different lentiviral-driven shRNAs (figure A, B and see online supplementary figure S2A–C). PaTu8988S cells grew as compact colonies and, upon GATA6 silencing, acquired a spindle-like shape and showed increased scattering. Furthermore, E-cadherin was downregulated and vimentin was upregulated (figure 1A, B), all features suggesting an EMT. Similarly, GATA6-silenced A13B cells showed lower E-cadherin levels (see online supplementary figure S2B, S2D) and GATA6-silenced SK-PC-1 cells showed increased vimentin levels (see online supplementary figure S2D). Despite the partially different cell-specific effects of GATA6 silencing (likely dependent on the extent of downregulation and the genetic background), we observed a common convergence towards EMT. Consistently, GATA6 overexpression in L3.6pl PDAC cells—displaying a looser growth pattern—resulted in the formation of compact colonies, reduced scattering, upregulated E-cadherin expression and downregulated vimentin (figure 1C–D and see online supplementary figure S2F). These findings support a mesenchymal–epithelial transition (MET) and demonstrate that GATA6 maintains the canonical epithelial phenotype in PDAC cells.

10.1136/gutjnl-2015-311256.supp1 Supplementary data

### GATA6 inhibits invasion in vitro and cell dissemination in vivo

**Figure 2:**
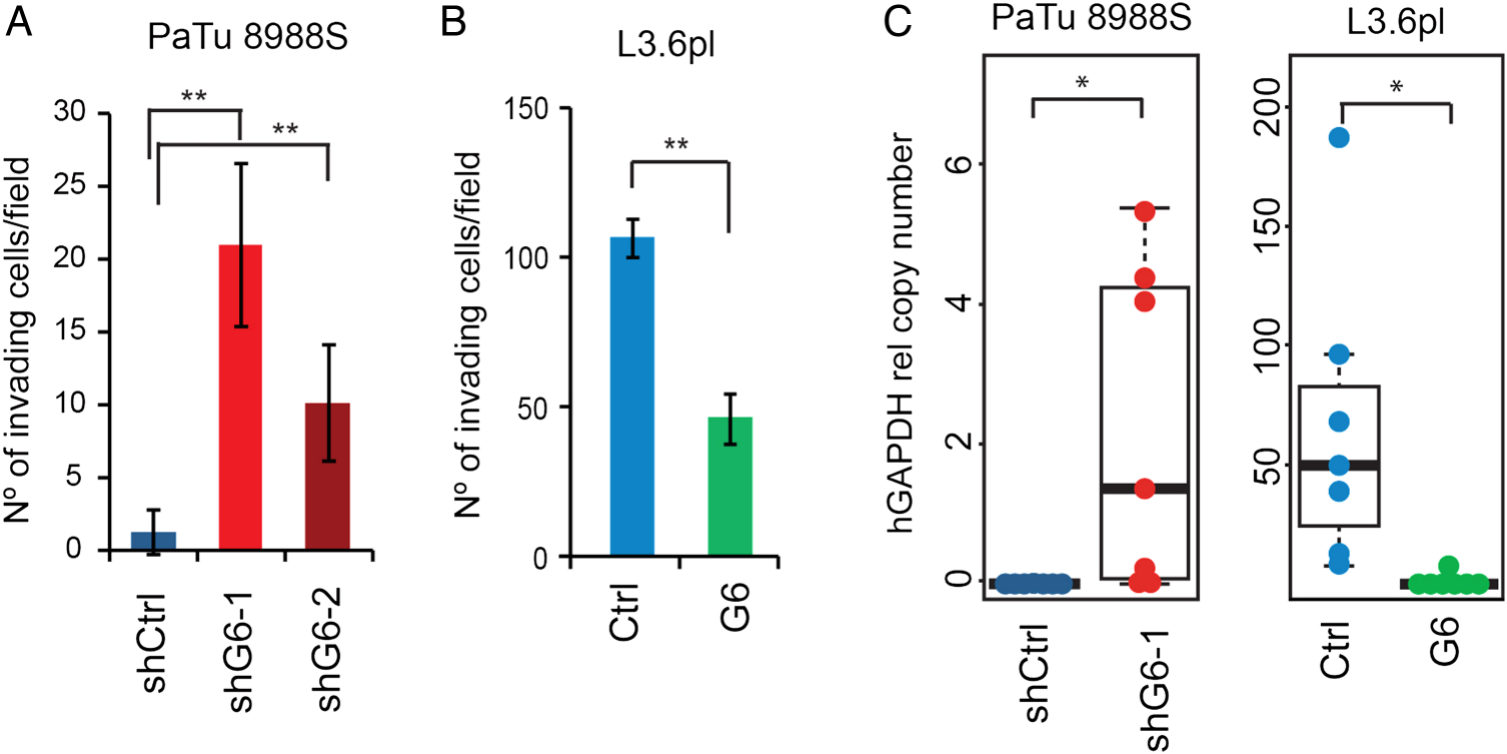
GATA6 inhibits invasion in vitro and cell dissemination in vivo. (A) Quantification of the in vitro invasiveness of PaTu8988S cells infected with the indicated constructs, measured as number of invading cells per microscopic field (×20 magnification). Data are mean±SEM of at least three independent experiments; **p<0.01. (B) Quantification of the in vitro invasiveness of L3.6pl cells infected with the indicated constructs. Data are mean±SEM of at least three independent experiments; **p<0.01. (C) Quantification of the metastatic burden in the liver of nude mice after intrasplenic injection of the indicated cells, measured by qPCR with human-specific primers detecting *HPRT*; *p<0.05.

EMT plays an important role in tumour progression and spreading^20^ ^21^ and is associated with the outcome in patients with PDAC.^22^ Consistently, GATA6-silenced PaTu8988S and SK-PC-1 cells displayed increased capacity to invade in vitro (figure 2A and see online supplementary figure S2E), while invasiveness was reduced in L3.6pl cells overexpressing GATA6 (figure 2B). To assess the contribution of GATA6 to tumour cell dissemination, we injected GATA6-silenced PaTu8988S and GATA6-overexpressing L3.6pl cells—and the respective control cells—into the spleen of athymic *Foxn1^nu^* mice and measured human gene expression in the liver by qPCR, an estimate of dissemination. GATA6 silencing in PaTu8988S cells significantly increased their capacity to reach the liver (p=0.048), while GATA6 overexpression in L3.6pl cells had the opposite effect (p=0.032) (figure 2C).

These data suggest that, through the regulation of EMT/MET, GATA6 might inhibit the acquisition of metastatic potential in PDAC cells. Furthermore, GATA6 was expressed at comparable levels in primary tumours (n=145) and adjacent normal pancreas (n=46) included in a recently published dataset,^23^ while it was significantly reduced in metastases (n=61) (p<0.001, see online supplementary figure S3), consistent with an antimetastatic role for GATA6 in patients.

### GATA6 blocks EMT directly and indirectly

EMT is mainly controlled by SNAI, ZEB and TWIST transcription factors, repressing E-cadherin expression and epithelial differentiation,^20^ while few positive regulators of the epithelial programme are known.

**Figure 3:**
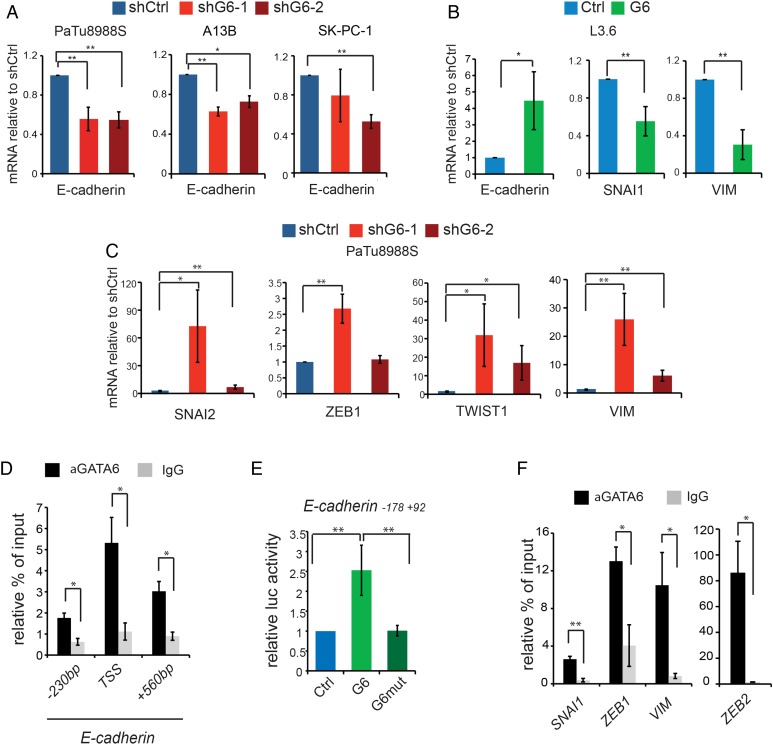
GATA6-dependent direct and indirect transcriptional regulation of epithelial–mesenchymal transition (EMT). (A) Expression of E-cadherin in PaTu8988S, A13B and SK-PC-1 cells infected with the indicated shRNA constructs, detected by RT-qPCR. (B) Expression of E-cadherin, SNAI1 and VIM (vimentin) in L3.6pl cells infected with the indicated constructs, measured by RT-qPCR. (C) Expression of SNAI2, ZEB1, TWIST1 and VIM in PaTu8988S cells infected with the indicated constructs, measured by RT-qPCR. (D) GATA6 binding to the indicated regions of the E-cadherin promoter detected by ChIP-qPCR in PaTu8988S cells. (E) Luciferase-based reporter assay showing the activity of an *E-cadherin* reporter in HEK293 cells transfected with empty vector (blue) or with vectors expressing either wild-type (light green) or mutated (dark green) GATA6. (F) GATA6 binding to the promoters of the indicated genes, detected by ChIP-qPCR in PaTu8988S cells. Data are presented as mean±SEM of at least three independent experiments. *p<0.05, **p<0.01 in all panels. ChIP-qPCR data are represented as % of input normalised against a negative control sequence, compared with binding of non-specific IgG; statistical significance is calculated for the enrichment of GATA6 binding to the region of interest, compared with the negative sequence.

E-cadherin mRNA was reduced in all GATA6-silenced cells analysed (figure 3A) and upregulated in GATA6-overexpressing L3.6pl cells (figure 3B). Furthermore, mRNA levels of SNAI2, ZEB1 and TWIST1 were upregulated in GATA6-silenced PaTu8988S cells, as were the levels of the mesenchymal marker vimentin (figure 3C). Accordingly, SNAI1 and vimentin mRNA levels were reduced in GATA6-overexpressing L3.6pl cells (figure 3B). These data suggest that GATA6 can regulate EMT-MET through the canonical pathway involving EMT-inducing transcription factors (EMT-TFs). The GATA6-dependent changes in EMT-TFs levels varied among different cell lines, suggesting convergence in the regulation of EMT-MET.

To further unravel how GATA6 regulates EMT, we determined its genome-wide distribution in PaTu8988S cells using ChIP-Seq. GATA6 occupied 26 248 genomic regions (FDR<0.01, see online supplementary dataset S1). The canonical GATAA sequence was the most enriched motif in the sequenced tags (see online supplementary figure S4A, E-value: 3.8e-350). GATA6 peaks were preferentially found (40%) within 1 kb from the transcription start site (TSS) of coding genes (see online supplementary figure S4B). ChIP-qPCR confirmed the ChIP-Seq results for a subset of genes (see online supplementary figure S4C).

A manual EMT-targeted analysis revealed two GATA6 peaks in the *E-cadherin* locus (see online supplementary figure S4D). One of them included the TSS and contained a non-canonical GATC sequence to which GATA3 binds in breast cancer cells.^24^ We confirmed GATA6 binding on this sequence and on the TSS (figure 3D). Wild-type GATA6—but not a DNA-binding mutant^17^—enhanced the activity of an *E-cadherin* promoter–reporter construct including the TSS (figure 3E), indicating direct transcriptional activation. Another peak is close to four canonical GATAA motifs; binding in the proximity of the first of them was confirmed by ChIP-qPCR (figure 3D). GATA6 also bound the promoter of multiple epithelial genes, including protocadherins, tight junction components (*CLDN1, CLDN4, CLDN7, OCLN, TJP1, TJP2, TJP3*), desmosomal proteins (*DSC2, DSC3, DSG2*), integrins, and keratins. We observed GATA6 binding to the promoter of *SNAI1* and *ZEB1,* and to the second intron of *ZEB2* (confirmed by ChIP-qPCR; figure 3F and see online supplementary figure S4D). GATA6 was also found in the promoter of *VIM* (coding for vimentin) and other mesenchymal genes (figure 3F and see online supplementary figure S4D). Gene-enrichment and functional annotation analysis (DAVID suite)^25^ on 5643 GATA6 peaks located <1 kb from a TSS and with FDR<0.1% (see online supplementary table S1) revealed enrichment of ‘focal adhesion’, ‘tight junction’, and ‘regulation of actin cytoskeleton’ pathways. The TGFβ and ERBB pathways, involved in EMT and in PDAC,^10^ were also enriched. These results indicate that GATA6 has a broad direct proepithelial function and concomitantly inhibits the mesenchymal programme.

### GATA6 regulates the E-cadherin inducers FOXA1 and FOXA2

**Figure 4:**
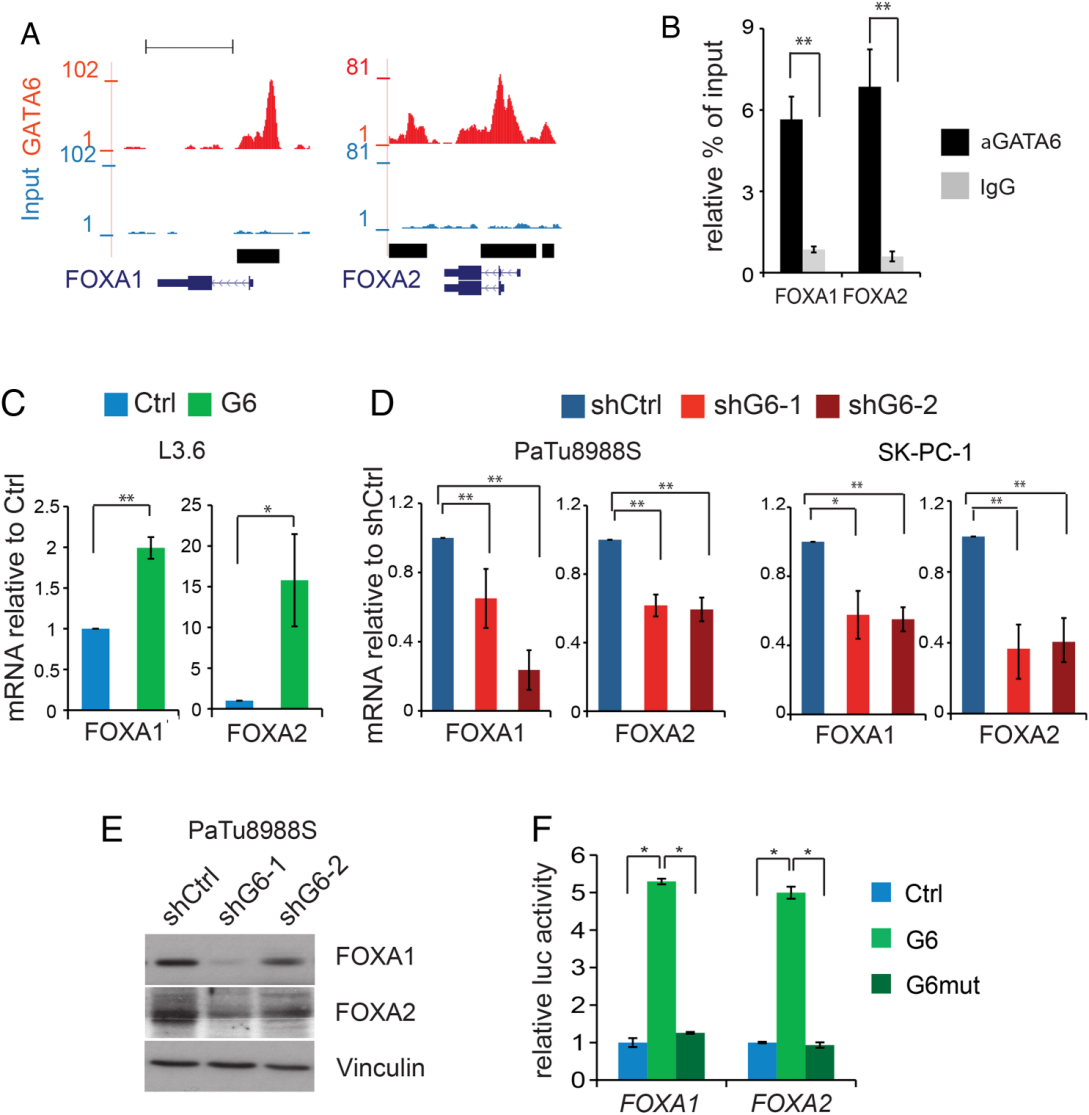
GATA6 directly activates the proepithelial transcription factors *FOXA1* and *FOXA2*. (A) Representation of ChIP-Seq peaks on *FOXA1* and *FOXA2* promoters. (B) GATA6 binding to the promoters of *FOXA1* and *FOXA2* detected by ChIP-qPCR in PaTu8988S cells. (C–D) Expression of FOXA1 and FOXA2 in L3.6 (C), PaTu8988S and SK-PC-1 (D) cells infected with the indicated constructs, measured by RT-qPCR. (E) Expression of FOXA1 and FOXA2 proteins in GATA6-silenced PaTu8988S cells. Vinculin was used as loading control. (F) Luciferase-based reporter assay showing the activity of *FOXA1* and *FOXA2* promoter reporters in HEK293 cells transfected with empty vector (blue) or with vectors expressing either wild-type (light green) or mutated (dark green) GATA6. In all the panels, data are presented as mean±SEM of at least three independent experiments; *p<0.05, **p<0.01. ChIP-qPCR data are represented as % of input normalised against a negative control sequence, compared with binding of non-specific IgG; statistical significance is calculated for the enrichment of GATA6 binding to the region of interest, compared with the negative sequence.

Among the few known E-cadherin transcriptional activators are FOXA1 and FOXA2,^26^ two important regulators of pancreatic development.^27^ Prominent GATA6 peaks in *FOXA1* and *FOXA2* suggested strong binding (figure 4A), confirmed at their TSS by ChIP-qPCR (figure 4B). FOXA1/2 mRNAs were upregulated in GATA6-overexpressing L3.6 cells (figure 4C) and repressed in GATA6-silenced PaTu8988S and SK-PC-1 cells (figure 4D); FOXA1/2 proteins were reduced in GATA6-silenced PaTu8988S cells (figure 4E). Furthermore, wild-type GATA6—but not the mutant—activated *FOXA1* and *FOXA2* promoter–reporter constructs (figure 4F). Interestingly, the FOXA DNA binding sequence was the second most enriched motif in the GATA6 ChIP-Seq (see online supplementary figure S4A), and we confirmed FOXA2 binding to a subset of GATA6 targets including both activated and repressed genes (see online supplementary figure S4E). Altogether, these data indicate that GATA6 activates transcription of E-cadherin, and possibly other targets, also indirectly through the induction of FOXA1 and FOXA2. GATA6 and FOXA1/2 thus cooperate in their proepithelial function. To assess the contribution of FOXA1/2 to GATA6-dependent functions, we silenced them individually in GATA6-overexpressing L3.6pl cells, but massive cell death precluded further analyses (not shown).

### GATA6 is lost in human PDAC, in association with loss of epithelial differentiation

**Figure 5:**
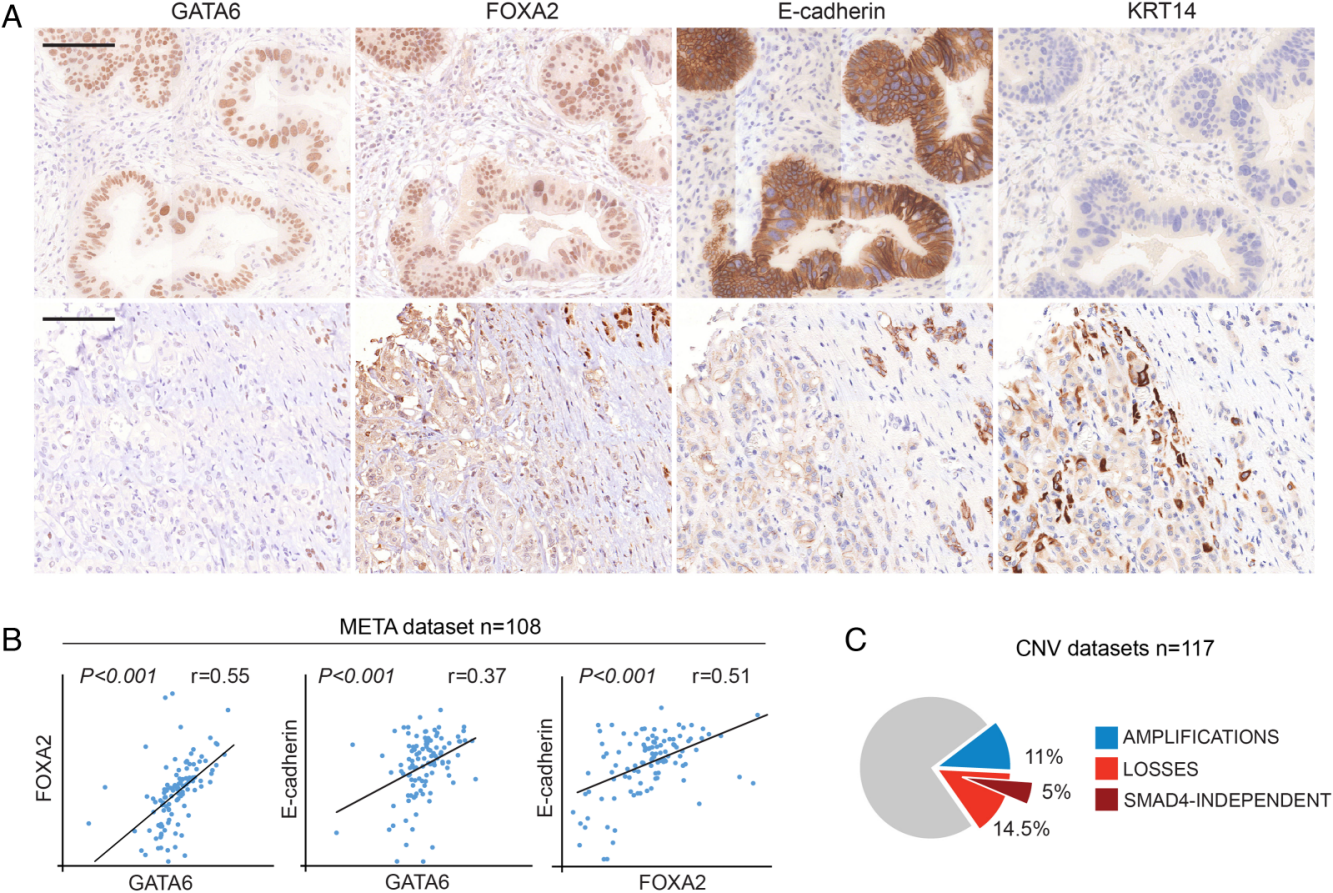
GATA6 loss in human pancreatic ductal adenocarcinoma (PDAC) is associated with altered differentiation. (A) Expression of GATA6, FOXA2, E-cadherin and KRT14 in two PDAC samples, detected by immunohistochemistry. Top: cells retaining GATA6 expression are FOXA2^high^, E-cadherin^high^ and KRT14^neg^; bottom: GATA6^neg^ cells are FOXA2^low^, E-cadherin^low^ and KRT14^pos^. Scale bar: 50 μm. (B) Scatter plots showing correlated expression of GATA6, FOXA2 and E-cadherin mRNA in the PDAC meta-dataset. (C) Proportion of tumours showing *GATA6* amplification (blue) or genomic loss (red) in the combined analysis of three PDAC gene copy number variation datasets. The percentage of *GATA6* losses that were independent from loss of *SMAD4* is represented in dark red.

We analysed GATA6, E-cadherin and FOXA2 by IHC in tumours (n=25) using 4 mm core tissue microarrays (TMA), allowing for detection of intratumour heterogeneity. GATA6 was lost broadly or focally in 4 (16%) and 12 cases (48%), respectively. E-cadherin was consistently low/mislocalised in all the GATA6^neg^ tumours and in areas of focal GATA6 loss. Likewise, FOXA2 was low in the GATA6^neg^ regions, supporting the relevance of the GATA6–FOXA2–E-cadherin axis in primary PDAC (figure 5A). In a meta-dataset of four published PDAC gene expression studies (META, n=108),^28–31^ we confirmed a positive correlation of GATA6, FOXA2 and E-cadherin mRNA levels (p<0.001 for all comparisons; figure 5B). Similar correlations were observed in an independent series (Moffitt, see online supplementary figure S5).^23^ FOXA1 expression did not correlate with GATA6, FOXA2 or E-cadherin (data not shown), suggesting that FOXA2 is the main GATA6 partner in PDAC.

Our observations suggest a tumour-suppressive role of GATA6 in human PDAC, concordant with our findings for mouse PDAC.^18^ This notion is at odds with the occurrence of *GATA6* amplifications in 10%–20% of PDACs,^15^ ^16^ ^32^ which led to the proposal that it is a PDAC oncogene. To solve this conundrum, we reanalysed *GATA6* gene copy number changes in three PDAC series (CNV, see online supplementary table S2):^32^ ^33^ ^34^ 13/117 (11%) tumours showed amplifications, but losses occurred at a similar rate (17/117, 14.5%) (figure 5C and see online supplementary table S2). *GATA6* is on 18q11, 28.7 Mb from *SMAD4*, which is frequently deleted in PDAC. *GATA6* and *SMAD4* were lost concomitantly in 11/17 cases and separately in 6/17 cases, suggesting that an independent selective pressure acts against *GATA6* in some PDACs (figure 5C and see online supplementary table S2). *GATA6* losses were confirmed in a subset (9/100) of PDAC recently reported by the Australian Pancreas Cancer Initiative.^35^

### Low GATA6 identifies a PDAC subtype with basal-like features

To gain insight into GATA6 function in PDACs, we compared the transcriptome of tumours belonging to the highest and lowest GATA6 expression quartiles in the PDAC meta-dataset (GATA6^high^ and GATA6^low^, n=27 for each group) and identified 495 genes upregulated or downregulated in GATA6^low^ versus GATA6^high^ with FDR<0.01 (see online supplementary dataset S2). Gene sets induced in basal-like (BAS-L) and suppressed in luminal-like breast cancers were enriched among genes upregulated in GATA6^low^ tumours (see online supplementary table S3 and figure S6).

Recently, a BAS-L subtype of bladder cancer was described carrying similarities with the corresponding breast cancer subtype, suggesting that poorly differentiated carcinomas of distinct origin might converge to a similar molecular phenotype. Hierarchical clustering of the meta-dataset samples according to a bladder cancer-defined 47-gene signature (BASE47)^36^ identified a BAS-L subgroup of PDAC (see online supplementary figure S7A).

Basal keratins are expressed in a subset of PDACs but are undetectable in normal pancreas.^12^ ^37^ Using the TMAs described earlier, KRT14 was found in the GATA6^neg^ regions of 7/16 PDACs, while it was absent from GATA6^high^ regions (figure 5A). Consistently, GATA6 was significantly lower in BAS-L tumours (p<0.001, see online supplementary figure S7B), and GATA6-silenced PaTu8988S cells ectopically expressed the basal keratins KRT5 and KRT14 (figure 1B). Furthermore, ChIP-Seq data showed GATA6 binding to the promoter of genes belonging to multiple published basal-related signatures,^36^ ^38^ ^39^ some of which were also regulated in the RNA-Seq experiment (see online supplementary dataset S3). Altogether, these data suggest that GATA6 participates in the regulation of the BAS-L transcriptional programme and that basal-like PDACs are GATA6^low^.

### Low GATA6 expression predicts poor survival and distinct response to adjuvant chemotherapy in patients with PDAC

**Figure 6:**
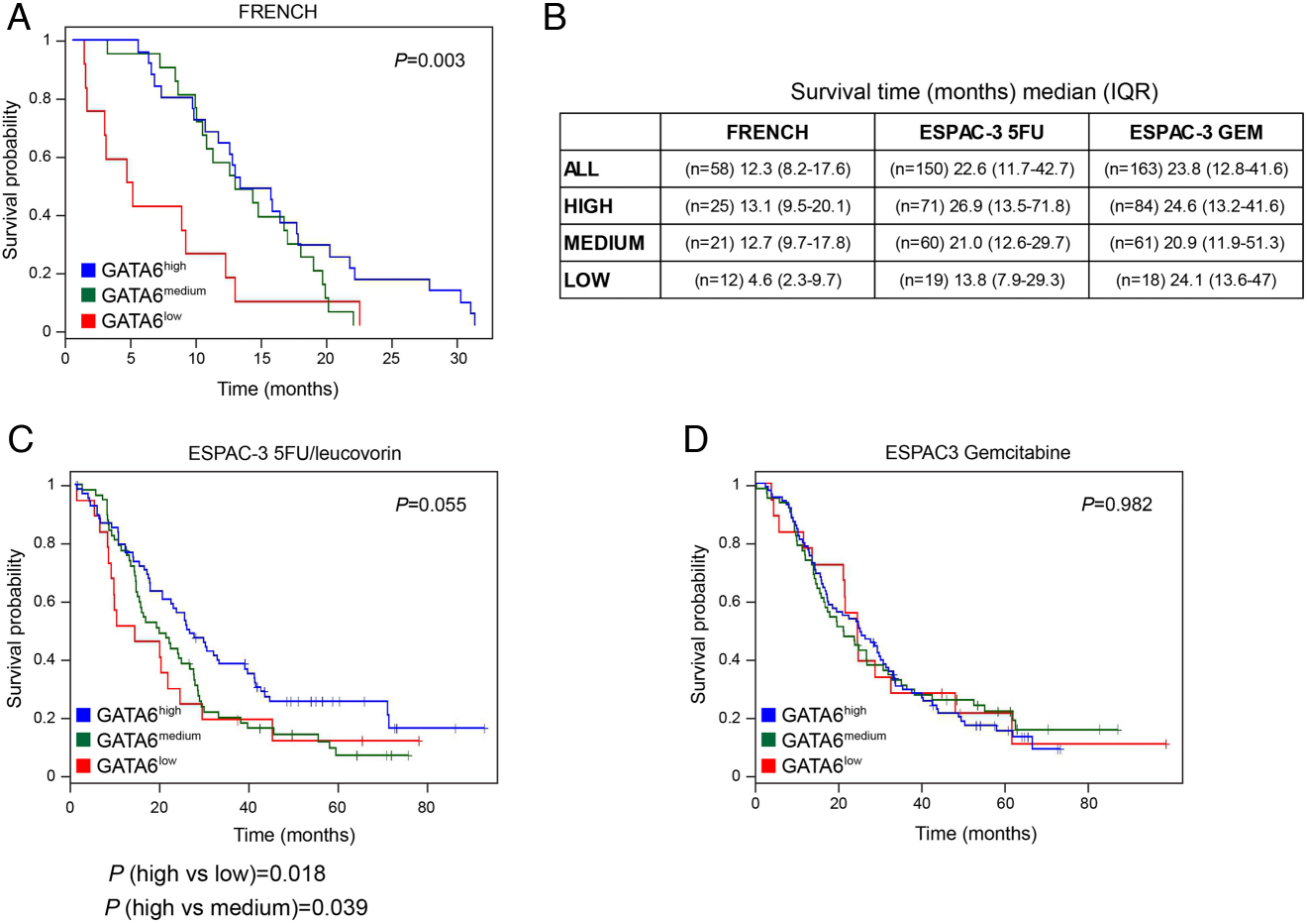
GATA6 expression is associated with outcome and with response to adjuvant therapy. (A) Kaplan–Meier plot of the overall survival for patients included in the French series. (B) Median survival of patients included in the French series, classified according to GATA6 level. The value of p=0.003 calculated with Mann–Whitney U test. (C) Kaplan–Meier plot of the overall survival for patients included in the 5-fluorouracil (5-FU)/leucovorin arm of the ESPAC-3 trial. (D) Kaplan–Meier plot of the overall survival for patients included in the gemcitabine arm of the ESPAC-3 trial.

Patients with BAS-L bladder and breast tumours have worse outcome and a distinct response to therapy.^36^ ^38^ ^39^ To assess the impact of GATA6 loss on patient survival, we analysed a series of 58 patients from whom xenografts were established and transcriptome data were available. Patients were categorised based on GATA6 expression values in three groups (<500, 500–1000, >1000). In this exploratory series, GATA6 levels were not significantly associated with the clinical–pathological variables considered (see online supplementary table S4). The survival of patients with GATA6^medium^ and GATA6^high^ tumours was similar (12.7 vs 13.1 months, respectively) and significantly longer than those with GATA6^low^ tumours (4.6 months, p=0.003) (figure 6A,B). Low GATA6 expression was associated with significantly increased death risk both in the univariate analysis (HR=5.39, 95% CI 2.3 to 12.9; p<0.001) and in the model adjusted by age and gender (HR=3.77, 95% CI 1.74 to 8.17; p=0.001) (table 1).

**Table 1:** Results of the univariate analysis of survival at 3, 6, 12 and 24 months (French series)

	Survival (months)				Risk of mortality					
	3	6	12	24						
Variable	n	n	n	n	HR crude	95% CI	p Value	HR adjusted	95%CI	p Value
GATA 6 (low/medium/high)										
High (N=25)	25	24	16	4	1.00			1.00		
Medium (N=21)	20	20	12	12	0.97	0.4 to2.6	0.943			
Low (N=12)	7	5	3	3	5.39	2.3 to 12.9	<0.001	3.77	1.74 to 8.17	0.001

To further explore the relationship between GATA6 expression and patient outcome, and its predictive value, we analysed TMAs from patients included in the ESPAC-3 trial.^7^ Using a histoscore based on the proportion of reactive cells and staining intensity, GATA6 expression was low/undetectable in 37/313 (11.8%) tumours. GATA6 levels were associated with tumour grade (p=0.005) but not with other clinical–pathological variables (see online supplementary table S5). Both treatment arms were well balanced regarding patient demographics (see online supplementary table S6). In the 5-FU/leucovorin arm, patients with GATA6^low^ or GATA6^medium^ tumours survived significantly less than patients with GATA6^high^ tumours (p values 0.018 and 0.039, respectively) (figure 6C). By contrast, GATA6 expression was not associated with survival in the gemcitabine arm (figure 6D). In the univariate analysis, GATA6 levels showed a marginally significant association with outcome, exclusively among patients receiving 5-FU/leucovorin (p=0.057) (table 2, see online supplementary table S7). Multivariable analysis did not reveal additional correlations (see online supplementary table S8). Furthermore, KRT14 expression was not predictive of outcome (see online supplementary table S7). These results support the notion that patients with GATA6^low/medium^ tumours might benefit less from treatment with 5-FU/leucovorin than from treatment with gemcitabine.

**Table 2:** Results of the univariate analysis of survival (ESPAC-3)

	Risk of mortality		
	5-FU/leucovorin	Gemcitabine	Total
GATA 6	N=150	N=163	N=313
High	1.00	1.00	1.00
Medium	1.49 (1.01–2.20)	0.97 (0.67–1.39)	1.19 (0.91–1.55)
Low	1.73 (0.99–3.03)	0.99 (0.56–1.72)	1.27 (0.86–1.89)
	Wald χ^2^ =5.72, p=0.057	Wald χ^2^ =0.04, p=0.982	Wald χ^2^ =2.38, p=0.304

5-FU, 5-fluorouracil.

**Figure 7:**
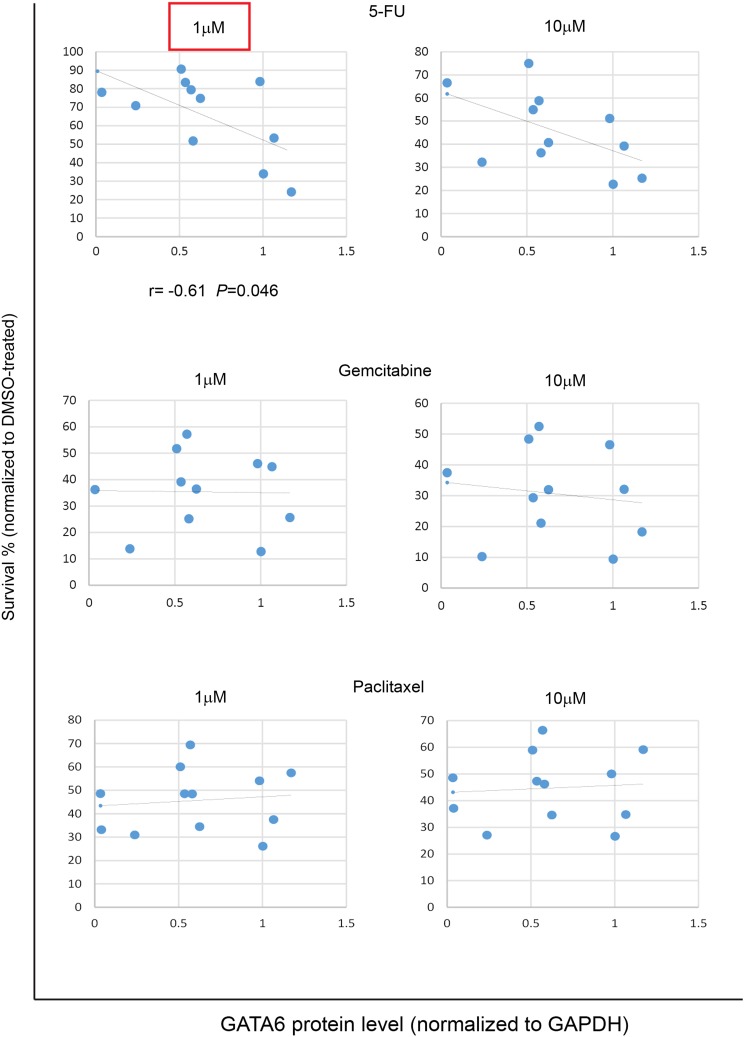
GATA6 expression negatively correlates with sensitivity to 5-fluorouracil (5-FU) in pancreatic ductal adenocarcinoma (PDAC) cells. Scatter plots showing cell survival upon treatment with the indicated doses of 5-FU (top), gemcitabine (middle) and paclitaxel (bottom), plotted against GATA6 protein level. Red square indicates significant correlation. Survival was normalised against DMSO-treated cells. Data are presented as the average value of at least three independent experiments.

We treated a panel of 11 primary cell lines established from patient-derived xenografts (TKCC cells, see online supplementary figure S8A)^35^ with increasing doses of 5-FU, gemcitabine or paclitaxel, and monitored the cytotoxic effect of the drugs. GATA6^low^ cells showed significantly lower sensitivity to 1 μM 5-FU (r=−0.61, p=0.046) and a consistent tendency to lower sensitivity to all other 5-FU concentrations (figure 7 and see online supplementary figure S8B), while no correlation was observed with gemcitabine or paclitaxel, regardless of the concentrations used (figure 7 and see online supplementary figure S8C,D). These findings support the selective association of GATA6 levels with 5-FU response as observed in the patients included in ESPAC-3 trial.

To investigate whether GATA6 has a causative role in the response to 5-FU, we knocked it down in PaTu8988S cells, as well as in the 5-FU sensitive, GATA6^high^, TKCC18 and TKCC19 cells, and we overexpressed it in L3.6pl cells and in the 5-FU resistant, GATA6^low^, TKCC9, TKCC15 and TKCC26 cells. However, we did not observe significant changes in the sensitivity of these cells to 5-FU, gemcitabine or paclitaxel (see online supplementary figure S9,S10, and data not shown).

## Discussion

An improved understanding of PDAC biology and tumour taxonomy should leverage on the exploitation of available therapies. Here, we provide important evidence in these directions. We extend prior data indicating GATA6 as a hallmark of tumour differentiation, provide strong evidence that it regulates the epithelial phenotype through novel mechanisms and show its potential as a marker for patient stratification.

GATA6 has a proepithelial and anti-EMT function in PDAC, and it does so through a unique mechanism, involving both the activation of epithelial genes and the concomitant repression of mesenchymal genes. Furthermore, the action of GATA6 is dual: direct—through the regulation of epithelial and mesenchymal genes—and indirect—through the regulation of proepithelial and promesenchymal transcription factors. To our knowledge, GATA6 is the first EMT regulator with such properties. GATA6 blocks dedifferentiation and the acquisition of metastatic properties in lung adenocarcinoma cells,^40^ but the underlying mechanisms had not been elucidated. Here, we show that the same is true for PDAC cells, where GATA6 downregulation increased tumour cell dissemination. Consistently, in the ESPAC-3 patient cohort, low GATA6 expression correlated with moderate/poor tumour grade. Although no significant correlation was observed with lymph node status or local invasion, the GATA6 histoscore showed a tendency to be lower in patients that were positive for either parameter. These observations, together with our in vitro and in vivo data, further support that GATA6 plays a role in inhibiting tumour spreading, although other factors appear to be involved.

Of note, GATA6-silenced PDAC cells showed reduced proliferation (not shown), as previously reported,^15–17^ consistent with the observation that EMT is associated with slower proliferation and reduced tumour growth.^41^ Proliferation was likewise reduced in GATA6-overexpressing L3.6pl cells (not shown), suggesting a more complex function for GATA6. Distinct genetic (ie, SMAD4 status, see online supplementary table S9) and epigenetic landscapes might account for the discrepancy with the previous reports.^17^

GATA6 also represses a basal-like transcriptional programme similar to the one described in breast and bladder^36^ ^38^ ^42^ ^43^ and, more recently, in PDAC.^23^ Loss of canonical differentiation was previously associated with low GATA6, both in PDAC^12^ and in lung cancer.^40^ Furthermore, a GATA6-overexpression signature was enriched in the classical PDAC subtype described recently.^23^ However, a mechanistic explanation was completely missing. Our work supports a causal role for GATA6 in repressing this BAS-L programme in PDAC. Interestingly, a cell population with a BAS-L phenotype is present in normal multilayered epithelia, such as breast, bladder and lung, but not in the single-layered pancreatic epithelium. Therefore, the emergence of a basal-related programme does not necessarily reflect the cell of origin of the tumour, as it was proposed, but it might represent a common ‘low-energy’ state for multiple tumour epithelial cell types. Alternatively, the BAS-L phenotype in PDAC might represent a transition to an ectopic differentiation programme, which could be defined as an ‘epithelial-to-epithelial transition’ (ET^2^). ET^2^ differs from the activation of lineage-preserved ectopic differentiation programmes, such as the gastric phenotype observed in PDAC precursors,^44^ also repressed by GATA6 in mice.^18^ In the pancreas, the basal programme defined by the ET^2^ concept does not represent a developmental feature. While ET^2^ may herald a full-blown EMT during tumour progression, these processes seem to be independent in lung adenocarcinoma, where GATA6^low^ BAS-L tumours lack EMT features.^40^ More investigations are required to assess the putative sequence from ET^2^ to EMT in other tumour types and a more general role of the GATA and FOXA protein families.

Concertedly, these findings contribute to explain the conundrum generated by observations supporting that GATA6 acts as oncogene in PDAC; yet, patients with GATA6-low tumours have worse outcome.

Sequentially regulated EMT and MET are required for efficient tumour spreading.^45^ ^46^ GATA6 regulates both processes; therefore, we hypothesise that the genetic context, as well as the microenvironment, might select for loss versus gain of GATA6 expression. Multiple evidences point to context-dependent functions: GATA6 favours EMT in vivo in *Drosophila melanogaster* and in vitro in MDCK cells^47^ and is required for the tumourigenic activity of *Apc* loss in the mouse colon.^48^ The different outputs might depend on the levels/localisation of other transcriptional regulators and coactivators/repressors and the epigenetic landscape. We propose that *GATA6* belongs to a new type of cancer genes whose effect can be oncogenic or tumour-suppressive depending on the cellular/genomic context.

GATA6 loss leads to EGFR pathway activation in PDAC cells and in mouse PDAC,^18^ suggesting a predictive, or causal, role for GATA6 in treatment response in patients. To explore this notion, we analysed samples from patients included in ESPAC-3, a randomised adjuvant trial comparing 5-FU/leucovorin and gemcitabine.^7^ The ESPAC-3 trial showed that both treatments had comparable effects on overall survival. We show that patients with GATA6^low^ tumours do not benefit from adjuvant 5-FU/leucovorin and have a significantly lower survival than similarly treated patients with GATA6^high^ tumours. By contrast, GATA6 expression was not associated with the response to gemcitabine. Altogether, these results point to GATA6 as a predictive marker for patient stratification. Given the antitumour activity of FOLFIRINOX in patients with PDAC, it will be important to determine whether GATA6 also predicts response to this drug combination. In addition, the joint analysis of hENT and GATA6 expression may show enhanced predictive ability.

The mechanism underlying the lack of response to 5-FU/leucovorin observed in GATA6^low^ tumours is still to be elucidated. The appearance of EMT features in 5-FU-resistant cells in vitro has been reported in various solid tumours, including PDAC,^49^ ^50^ ^51^ but a cause–effect relationship is lacking. Modulation of GATA6 levels in TKCC cells did not change their sensitivity to chemotherapeutic drugs, suggesting that GATA6 is part of a molecular phenotype involved in drug response, but it is not its major driver.

In conclusion, we provide here a thorough mechanistic analysis of GATA6 function in PDAC cells, where it inhibits EMT, basality and dissemination, supporting its role as a PDAC tumour suppressor, further strengthened by the genomic losses that we and others observed, and by the hypermethylation of *GATA6* promoter described recently.^52^ Finally, we propose GATA6 as a valuable marker to guide patient treatment.

## Methods

### Cell lines

HEK293T and PDAC cells were cultured in DMEM supplemented with 10% FBS and NaPyr, in standard conditions (37°C, 20% O_2_, 5% CO_2_), except for L3.6pl cells, which were cultured in RPMI with 10% serum. Mutational profile of the cells used is available in online supplementary table S9. We obtained HEK293T cells from ATCC, A13B from C. Iacobuzio-Donahue (Memorial Sloan Kettering, New York, USA), L3.6pl cells from C. Heeschen (CNIO, Madrid, Spain) and PaTu8988 S from M. Buchholz (University of Marburg, Germany). TKCC primary cell lines were established as described.^35^ All remaining PDAC cells were previously available in the laboratory.

### Cytotoxicity assays

Cells were seeded at low density (5000 cells/well) in 96-well plates and treated with either DMSO or increasing concentrations of 5-FU (1 nM–100 μM, SIGMA-Aldrich), gemcitabine (1 nM–100 μM, SIGMA-Aldrich) or paclitaxel (100 pM–10 μM, SIGMA-Aldrich). After 72 hours, cells were fixed with methanol and stained with crystal violet. Crystal violet was extracted with 1% SDS, and absorbance was measured at 595 nm.

### Plasmids, transfection and infection

Lentiviral vectors expressing non-targeting and GATA6-targeting shRNAs were purchased from SIGMA-Aldrich (MISSION shRNA). pcDNA3 plasmids containing human wild-type and mutated GATA6 cDNA were described earlier.^17^ GATA6 cDNA was cloned into the GFP-expressing FG12 lentiviral vector for overexpression in PDAC cells. Reporter plasmids containing Foxa1 and Foxa2 promoters were a generous gift of Dr RJ Matusik (Vanderbilt University, Tennessee, USA). Reporter plasmid containing the E-cadherin promoter was a generous gift of Dr A Nieto (Institute for Neurosciences, Alicante, Spain). Virus-packaging HEK293T cells were transfected with standard calcium phosphate protocol, supernatant was collected 48 hours after transfection, filtered and used to infect PDAC cells. Successfully infected cells were selected either with puromycin or by FACS-sorting.

### IHC and immunofluorescence

Sections were incubated with primary antibodies (see online supplementary table S10). HRP-conjugated secondary antibodies were from DAKO. DAB+ (3,3-diaminobenzidine tetrahydrochloride plus) was used as chromogen and nuclei were counterstained with haematoxylin. For immunofluorescence (IF) staining, Alexa-conjugated secondary antibodies from Invitrogen were used and nuclei were counterstained with 4′-6-diamidino-2-phenylindole (DAPI). Images were pseudocoloured using LEICA Application Suite.

### Gene expression analyses

Total RNA was extracted from cells using Trizol (SIGMA-Aldrich) according to manufacturer's instructions, treated with DNase I (Ambion DNA-free kit, Invitrogen) and converted to cDNA using TaqMan reverse transcription reagents (Applied Biosystems). Quantitative PCR was performed using SYBR-green mastermix (Applied Biosystems and Promega) and run in a Prism 7900 HT instrument (Applied Biosystems). Primers were designed using Primer3Plus, and reactions were done in triplicate. All quantifications were normalised to endogenous HPRT, using the standard ΔΔCt method. Primer sequences are provided in online supplementary table S11.

### Protein analysis

Protein extracts were prepared in Laemmli buffer and sonicated. SDS–PAGE–western blotting was done using standard protocols.^53^ Primary antibody information is provided in online supplementary table S11.

### Matrigel invasion assay

Transwells (BD Falcon, 0.8 μm) were coated with BD Matrigel. Cells (10^5^) were seeded onto Matrigel in serum-free DMEM and were allowed to invade towards DMEM with 10% FBS. Invading cells were fixed with PFA after 24 hours (L3.6pl) or 72 hours (PaTu8988S), nuclei were stained with DAPI and counted on a fluorescent microscope. The number of invading cells/field was normalised by the number of cells seeded in parallel in a separate well.

### Luciferase assay

HEK293T cells were transfected with E-cadherin, Foxa1 or Foxa2 reporter plasmids, together with a GFP-expressing plasmid. At the same time, empty pcDNA3 (Invitrogen), or pcDNA3 containing either wild-type or mutant GATA6 cDNA were introduced. Luciferase activity was measured with a luminometer, using a commercial luciferin solution (Promega) as a substrate. Values were normalised for transfection efficiency by checking GFP levels using western blotting.

### ChIP

Cells were cross-linked with 1% formaldehyde for 15 min at room temperature, harvested in lysis buffer (2×10^7^ cells/mL) and sonicated in a Covaris instrument (shearing time 30 min, 20% duty cycle, intensity 10, 200 cycles per burst, 30 s per cycle) in 2 mL. ChIP was performed using anti-GATA6 R&D AF1700 antibody, following a standard protocol.^54^ Independent chromatin immunoprecipitates were used for sequencing and for ChIP-Seq validation, using qPCR (primers are listed in online supplementary table S7).

### In vivo dissemination assay

Xenografts were performed as described.^19^ Briefly, 5×10^4^ cells were resuspended in 50 μL of PBS and injected into the spleen of athymic *Foxn1^nu^* mice; 10 weeks later, livers were explanted and homogenised in Trizol for RNA extraction. Human-specific *HPRT* primers were used to quantify the presence of human cells. Mice were purchased from Charles River Laboratories and maintained at CNIO under standard conditions. All experiments were approved by the Animal Ethical Committee of Instituto de Salud Carlos III (Madrid, Spain) and performed in accordance with the guidelines for Ethical Conduct in the Care and Use of Animals as stated in The International Guiding Principles for Biomedical Research involving Animals, developed by the Council for International Organizations of Medical Sciences (CIOMS).

### Microarray data and GSEA analyses

Hierarchical clustering of the PDAC meta-dataset was performed with Genepattern (http://www.genepattern.broadinstitute.org). The dataset was row-centred and column-centred, and row-normalised and column-normalised. Differential gene expression and GSEA were performed with the corresponding module from the same online suite.

### Patients and samples

Detailed information is provided in the online supplementary material.

### Statistical analyses

Data are provided as mean±SEM. Statistical analysis was performed using two-tailed Student's t test or one-tailed Fisher's test, and significance was considered for p<0.05. All statistical analyses were performed using VassarStat.net and R. Detailed information on the statistical tests used for the analysis of clinical data is provided as online supplementary material.
