# Supplementary material for: GATA6 regulates EMT and tumour dissemination, and is a marker of response to adjuvant chemotherapy in pancreatic cancer
Source: Gut. 2016 Jun 20;66(9):1665–76. doi: 10.1136/gutjnl-2015-311256 (PMC5070637; doi:10.1136/gutjnl-2015-311256)

GATA6 regulates EMT and tumor dissemination, and correlates with response to adjuvant chemotherapy in pancreatic cancer

Paola Martinelli[1,2*], Enrique Carrillo-de Santa Pau[1], Trevor Cox[3,4], Bruno Sainz Jr[5], Nelson Dusetti[6], William Greenhalf[4], Lorenzo Rinaldi[1#], Eithne Costello[3], Paula Ghaneh[3,4], Núria Malats[7], Markus Büchler[8], Marina Pajic[9], Andrew V Biankin[9,12], Juan Iovanna[6], John Neoptolemos[3,4], Francisco X. Real[1,13*]

SUPPLEMENTARY MATERIAL

EXTENDED EXPERIMENTAL PROCEDURES

ChIP-Seq. After performing ChIP as described, both input pool (20ng) and individual sample (6ng) DNA, were resolved by electrophoresis and fractions of 50-250bp were taken. 1-3 ng of DNA fraction per sample was processed through subsequent enzymatic treatments of end-repair, dA-tailing, and ligation to adapters as in Illumina's "TruSeq DNA Sample Preparation Guide" (part # 15005180 Rev. C). Adapter-ligated libraries were completed by limited-cycle PCR with Illumina PE primers.The resulting purified DNA library was applied to an Illumina flow cell for cluster generation (TruSeq cluster generation kit v5) and sequenced on the Genome Analyzer IIx with SBS TruSeq v5 reagents by following manufacturer's protocols. Image analysis and per-cycle base calling was performed with Illumina Real Time Analysis software (RTA1.13). Conversion to FASTQ read format was performed with CASAVA-1.8 (Illumina). Sequence alignment to the reference genome (GRCh37/hg19, February 2009) was made with BWA version 0.5.9-r16 algorithm allowing up to 0/1 mismatches, using default settings. Only unique reads were kept for the analysis and ChIP-seq and Input alignments files were normalized to the same number of reads before the peak detection.

To visualize the data in the University of California Santa Cruz genome browser (http://genome.ucsc.edu) the reads were directionally extended to 300 bp and, for each base pair in the genome, the number of overlapping sequence reads was determined and averaged over a 10 bp window to create a wig file. ChIP-Seq data are deposited in GEO: GSE47537.

Sequences of the primers used for ChIP-qPCR are shown in Supplementary Table S7.

Peak finding and motif discovery. Significant peaks of GATA6 binding were identified with MACS (1) v1.4 using model fold =20,40 and the other settings by default. Peaks with a FDR<1 were selected for further analysis. PeakAnalyzer software was used to identify functional elements proximal to the GATA6 peaks. Motifs were identified with the MEME suite (2), using peaks with FDR<1, and then TOMTOM (3) was used to compare the identified motifs with known transcriptional motifs.

Genomic analysis of gene copy number changes in *GATA6* and *SMAD4*. CGH data from human primary pancreatic tumors were obtained from public datasets under the accession numbers E-MTAB-570, GSE25273, and GSE7599. Agilent aCGH data were normalized with Limma 3.8.2 package using "normexp" method for background correction (4) and "median" method for normalization within arrays (5). WaviCGH (6) was used in further analyses with "DNAcopy" method for segmentation (7) and "probability" for calling (8). *GATA6* and *SMAD4* were considered lost or gained when the probability of either of the two events was higher than 0.5.

PDAC meta-dataset generation and analyses. Four expression datasets generated using Affymetrix array platform HG133plus2 were downloaded from GEO (GSE32688, GSE22780, GSE16515, GSE15471, involving 67 normal pancreas and 108 tumor pancreas samples. All datasets were normalized at the same time using Frozen Robust Multi-Array Analysis (fRMA) (9), furthermore the barcode algorithm was used to estimate whether the genes of interest were expressed or not in the different samples (10). Statistical analyses were performed using R version 2.14.1., the Mann–Whitney–Wilcoxon test was performed to compare GATA6 expression between samples expressing, or not, KRT14. Pearson's correlation coefficient was calculated for GATA6-FOXA2, GATA6-E-cadherin, and FOXA2-E-cadherin comparisons.

Patient samples and xenografts. Patients from the French series were included in the Paoli-Calmettes Institute clinical trial number 2011-A01439-32. Three expert clinical centers collaborated to the project after approval by the ethics review boards (approval number 11-61). Patient informed consent forms were collected and registered in a central database. The tumor tissue used for xenograft development was deemed excess to that required for the patient's diagnosis. Two types of samples were obtained, namely Endoscopic Ultrasound-Guided Fine-Needle Aspiration (EUS-FNA) biopsies from patients with unresectable tumors and a tumor piece from patients undergoing surgery. Pathology reports were obtained for each patient sample. All samples were anonymized. Histopathologic evaluation was performed on 5-μm H&E-stained sections of patient tumors and xenografts under a light microscope.

Each sample obtained from EUS-FNA was mixed with 100 µl of Matrigel (BD Biosciences) and injected in the upper right flank of a nude mouse (Swiss Nude Mouse Crl: NU(Ico)-Foxn1nu, Charles River Laboratories). Each sample derived from surgery resection was fragmented, mixed with 100 µl of Matrigel (BD Biosciences) and implanted with a trocar (10 Gauge, Innovative Research of America, Sarasota, FL) in the subcutaneous right upper flank of an anesthetized and disinfected mouse. When tumors reached 1 cm^3, mice were sacrificed and tumors were removed. The study on animals was approved by the "Plateforme of Stabulation et d'Expérimentation Animale" (PSEA), Scientific Park of Luminy, Marseille (11).

Gene expression microarrays. For RNA extraction, xenografted tumors were removed from mice at passage 2 and immediately placed in cold guanidinium thiocyanate (4 M), followed by protein denaturation and RNAs extraction according to Chirgwin's protocol (Chirgwin et al., 1979). Total RNA (1.0 µg) was reverse transcribed for hybridization to the human oligonucleotide array Human Gene 2.0 (Genechip, Affymetrix). Arrays were processed using the Affymetrix GeneChip Fluidic Station 450 (protocol EukGE-WS2v5_450) and scanned using a GeneChip Scanner 3000 G7 (Affymetrix). The GeneChip Operating Software (Affymetrix GCOS v1.4) was used to obtain chip images and for quality control. Background substraction and normalization of probe set intensities were performed using the method of Robust Multiarray Analysis (RMA). PCA analysis was performed using Genomics Suite software (Partek). Gene set enrichment analysis (GSEA) was performed on the Broad Institute Platform and statistical significance (false discovery rate, FDR) was set at 0.25. Microarray analysis was performed by the CRCHUL (CHUQ) Gene Expression Platform, Quebec, Canada.

All array data are available at National Center for Biological Information (NCBI) Gene Expression Omnibus (GEO) omnibus GSE55513

Statistical analyses on clinical data. The normality of the distribution of GATA6 expression values was assessed. For the French series, three categories were defined: low (<500), medium (500-1000), and high (>1000). Associations between demographics and clinico-pathological parameters and GATA6 categories were assessed using the Pearson's chi-squared test or Fisher's exact test (for N< 5). Kaplan-Meier curves were estimated for the 58 patients with available follow-up data. Overall median survival time (interquantile range, IQR) was 12.3 (8.2 - 17.6). Unadjusted and sex- and age-adjusted Cox proportional hazard regression was used to estimate the hazard ratio (HR) and 95% confidence intervals (95%CI) to analyze the association between levels of GATA6 and survival.

Data summaries of patients included in the ESPAC-3 study are provided as frequency counts for categorical variables and median (IQR) for continuous variables. Low,

medium and high GATA6 categories were defined for GATA6 scores <100, 100-200, >200 respectively. Hypothesis tests were performed for categorical variables using the chi-square test or Fisher's exact test with significance taken as $P<0.05$. Both univariate and multivariate Cox proportional hazards models were fitted, the latter using a stepwise approach. Kaplan-Meier curves were calculated and differences between groups assessed using the log-rank test. All statistical analyses were performed using SAS v9.3 statistical software.

Supplementary references

1. Zhang Y, Liu T, Meyer CA, Eeckhoute J, Johnson DS, Bernstein BE, et al. Model-based analysis of ChIP-Seq (MACS). Genome Biol. 2008;9:R137.

2. Machanick P, Bailey TL. MEME-ChIP: Motif analysis of large DNA datasets. Bioinformatics. 2011;27:1696–7.

3. Gupta S, Stamatoyannopoulos JA, Bailey TL, Noble WS. Quantifying similarity between motifs. Genome Biol. 2007;8:R24.

4. Ritchie ME, Silver J, Oshlack A, Holmes M, Diyagama D, Holloway A, et al. A comparison of background correction methods for two-colour microarrays. Bioinformatics. 2007;23:2700–7.

5. Smyth GK, Speed T. Normalization of cDNA microarray data. Methods. 2003;31:265–73.

6. Carro A, Rico D, Rueda OM, Díaz-Uriarte R, Pisano DG. waviCGH: a web application for the analysis and visualization of genomic copy number alterations. Nucleic Acids Res. 2010;38:W182–7.

7. Olshen AB, Venkatraman ES, Lucito R, Wigler M. Circular binary segmentation for the analysis of array-based DNA copy number data. Biostatistics. 2004;5:557–72.

8. Van de Wie MA, Kim KI, Vosse SJ, van Wieringen WN, Wilting SM, Ylstra B. CGHcall: Calling aberrations for array CGH tumor profiles. Bioinformatics. 2007;23:892–4.

9. McCall MN, Bolstad BM, Irizarry RA. Frozen robust multiarray analysis (fRMA). Biostatistics. 2010;11:242–53.

10. McCall MN, Uppal K, Jaffee HA, Zilliox MJ, Irizarry RA. The gene expression barcode: Leveraging public data repositories to begin cataloging the human and murine transcriptomes. Nucleic Acids Res. 2011;39.

11. Duconseil P, Gilabert M, Gayet O, Loncle C, Moutardier V, Turrini O, et al. Transcriptomic Analysis Predicts Survival and Sensitivity to Anticancer Drugs of Patients with a Pancreatic Adenocarcinoma. Am J Pathol. American Society for Investigative Pathology; 2015;185:1022–32.

SUPPLEMENTARY DATASETS

Supplementary Dataset 1. Genome-wide analysis of GATA6 binding to the DNA (ChIP-Seq) in PaTu8988S cells.

Supplementary Dataset 2. Gene expression comparison between $GATA6^{low}$ and $GATA6^{high}$ PDACs.

Supplementary Dataset 3. Overlap between the BASE47 gene signature, the GATA6 ChIP-Seq, and RNA-Seq data in PaTu8988S shG6-1 versus shCtrl.

SUPPLEMENTARY TABLES

Supplementary Table 1. Gene-Enrichment and Functional Annotation of the genes with a GATA6 peak within 1kb from TSS and FDR<0.1%.

Supplementary Table 2. Details of the CNV analyses in PDAC samples

Supplementary Table 3. GSEA analysis on the genes differentially expressed in GATA6low versus GATA6high tumors (FDR<0.01).

Supplementary Table 4. Univariate analysis of the 59 patients from the xenografted series classified in three GATA6-dependent categories.

Supplementary Table 5. Association of GATA6 with the clinico-pathological characteristics of the patients included in the ESPAC-3 trial.

Supplementary Table 6. Demographics of the patients included in the ESPAC-3 trial.

Supplementary Table 7. Full univariate analysis of the patients included in the ESPAC-3 trial.

Supplementary Table 8. Multivariate analysis of the patients included in the ESPAC-3 trial.

Supplementary Table 9. Mutational status of the PDAC cell lines used in this study, plus Panc1 (used in a previous study about GATA6).

Supplementary Table 10. Primary antibodies used in this study.

Supplementary Table 11. Sequence of primers used in this study.

SUPPLEMENTARY FIGURE LEGENDS

Supplementary Figure 1. GATA6 expression in PDAC cells. (A) RT-qPCR analysis of GATA6 mRNA levels in a panel of PDAC cell lines, relative to HPRT. In red are the cell lines that were used in subsequent experiments. (B) Western blot showing the protein levels of GATA6, E-cadherin, and vimentin. Vinculin was used as loading control.

Supplementary Figure 2. GATA6 knock-down and overexpression. (A-C) Top: microphotographs showing the PDAC cell lines that were used for *GATA6* knock-down experiments. Bottom: GATA6 expression in A13B, PaTu8988S, SK-PC-1 infected with the indicated constructs, measured by RT-qPCR. (D) Expression of GATA6, E-cadherin, and vimentin detected by western blotting in total lysates from A13B and SK-PC-1 cells infected with the indicated constructs. GAPDH was used as a loading control. (E) Quantification of the *in vitro* invasiveness of SK-PC-1 cells infected with the indicated constructs. (F) Microphotograph of parental L3.6pl cells, used for GATA6 overexpression, and (bottom) mRNA levels of GATA6 in L3.6pl cells infected with the indicated constructs. Data are presented as mean±s.e.m. of at least three independent experiments; *$P<0.05$, **$P<0.01$.

Supplementary Figure 3. GATA6 expression is lower in metastases compared to primary tumors. Boxplot showing the expression of GATA6 in normal pancreas (norm, adjacent to PDAC), primary tumors (PDAC), and metastases (met) in the patients' series described by Moffitt et al. **$P<0.001$

Supplementary Figure 4. GATA6 ChIP-Seq validation and ChIP analysis of FOXA2 on GATA6 targets. (A) Top: Representative genomic distribution of GATA6 in a region of human chromosome 14, as displayed in the UCSC genome browser. Tag counts (top-red for GATA6-ChIP and blue for input) and called peaks (bottom) tracks are shown. Bottom: core matrices obtained for the most significantly enriched motifs found in the GATA6 ChIP-Seq peaks. (B) Distribution of GATA6 ChIP-Seq peaks according to their distance from the closest transcription start site (TSS). (C) GATA6 binding to the promoter region of the indicated genes detected by ChIP-qPCR in PaTu8988S cells, represented as % of input chromatin and compared with non-specific IgG binding. Data are shown as mean±s.e.m of at least three independent experiments; *$P<0.05$, **$P<0.01$. (D) Representation of the ChIP-Seq peaks found in the promoter of SNAI1, ZEB1, ZEB2, and VIM, as displayed in the UCSC genome browser. Scale bar: 5Mb. (E) FOXA2 binding to the promoter of the indicated genes, selected as GATA6 targets. UP and DOWN refers to the regulation of the

corresponding mRNAs in GATA6-silenced PaTu8988S cells. Data are presented as mean±s.e.m. of at least three independent experiments; *P<0.05, **P<0.01.

Supplementary Figure 5. GATA6 correlates with the epithelial phenotype in primary tumors. Scatter plots showing correlated expression of GATA6, FOXA2, and E-cadherin mRNA in the Moffitt series. P<0.001 for all the comparisons.

Supplementary Figure 6. GATA6^{low} tumors display upregulation of pathways associated with basal breast cancer. GSEA plots showing some of the genesets that were most enriched within the genes upregulated in GATA6^{low} tumors.

Supplementary Figure 7. Low GATA6 is detected in a BAS-L subtype of PDACs. (A) Heat map showing the expression of the BASE47 signature in a meta-dataset of 108 PDAC samples; clustering was performed with the Hierarchical Clustering tool of the GenePattern suite. (B) Boxplot showing the expression of GATA6 in the two groups classified by the BASE47 signature. **P<0.0001.

Supplementary Figure 8. GATA6^{low} cells are insensitive to 5-FU. (A) WB showing the expression of GATA6 in the TKCC cells. GAPDH was used as loading control. (B-D) Scatter plots showing cell survival upon treatment with the indicated doses of 5-FU (B), gemcitabine (C), and paclitaxel (D), plotted against GATA6 protein level. Red square indicates the dose/drug showing significant correlation. Survival was normalized against DMSO-treated cells. Data are presented as median value of at least three independent experiments.

Supplementary Figure 9. GATA6 modulation does not change drug sensitivity of PaTu8988S and L3.6pl cells. (A) Western blot showing the extent of GATA6 overexpression and knock-down. Vinculin was used as loading control. (B) Survival of the indicated cells after treatment with increasing doses of either 5-FU or gemcitabine.

Supplementary Figure 10. GATA6 modulation does not change drug sensitivity of TKCC cells. (A) Western blot showing the extent of GATA6 overexpression and knock-down. GAPDH was used as loading control. (B) Survival of the indicated cells after treatment with increasing doses of either 5-FU or gemcitabine.

Supplementary Tables

Supplementary Table 1

Gene Set Name	p-value	FDR q-value
KEGG_MAPK_SIGNALING_PATHWAY	0.00E+00	0.00E+00
KEGG_REGULATION_OF_ACTIN_CYTOSKELETON	0.00E+00	0.00E+00
KEGG_HUNTINGTONS_DISEASE	0.00E+00	0.00E+00
KEGG_PATHWAYS_IN_CANCER	0.00E+00	0.00E+00
KEGG_AXON_GUIDANCE	0.00E+00	0.00E+00
KEGG_INSULIN_SIGNALING_PATHWAY	0.00E+00	0.00E+00
KEGG_CELL_CYCLE	0.00E+00	0.00E+00
KEGG_UBIQUITIN_MEDIATED_PROTEOLYSIS	0.00E+00	0.00E+00
KEGG_P53_SIGNALING_PATHWAY	0.00E+00	0.00E+00
KEGG_RIBOSOME	0.00E+00	0.00E+00
KEGG_CHRONIC_MYELOID_LEUKEMIA	0.00E+00	0.00E+00
KEGG_NEUROTROPHIN_SIGNALING_PATHWAY	1.11E-16	1.59E-15
KEGG_LYSOSOME	1.11E-16	1.59E-15
KEGG_ENDOCYTOSIS	2.22E-16	2.95E-15
KEGG_ALZHEIMERS_DISEASE	3.33E-16	4.13E-15
KEGG_PROSTATE_CANCER	1.44E-15	1.68E-14
KEGG_FOCAL_ADHESION	1.78E-15	1.94E-14
KEGG_WNT_SIGNALING_PATHWAY	2.78E-15	2.87E-14
KEGG_RENAL_CELL_CARCINOMA	3.00E-15	2.93E-14
KEGG_ERBB_SIGNALING_PATHWAY	4.00E-15	3.72E-14
KEGG_SMALL_CELL_LUNG_CANCER	7.11E-15	6.29E-14
KEGG_APOPTOSIS	4.03E-14	3.41E-13
KEGG_FC_GAMMA_R_MEDIATED_PHAGOCYTOSIS	2.42E-13	1.95E-12
KEGG_GLIOMA	8.30E-13	6.43E-12
KEGG_PANCREATIC_CANCER	1.22E-12	9.08E-12
KEGG_T_CELL_RECEPTOR_SIGNALING_PATHWAY	2.18E-12	1.56E-11
KEGG_OOCYTE_MEIOSIS	3.11E-12	2.14E-11
KEGG_PURINE_METABOLISM	7.90E-12	5.25E-11
KEGG_PARKINSONS_DISEASE	9.35E-12	5.99E-11
KEGG_SPLICEOSOME	9.85E-12	6.11E-11
KEGG_ADHERENS_JUNCTION	1.04E-11	6.22E-11
KEGG_RNA_DEGRADATION	1.77E-11	1.01E-10
KEGG_DNA_REPLICATION	1.79E-11	1.01E-10
KEGG_AMINO_SUGAR_AND_NUCLEOTIDE_SUGAR_METABOLISM	2.83E-11	1.46E-10
KEGG_LYSINE_DEGRADATION	2.83E-11	1.46E-10
KEGG_NUCLEOTIDE_EXCISION_REPAIR	2.83E-11	1.46E-10
KEGG_N_GLYCAN_BIOSYNTHESIS	8.67E-11	4.36E-10
KEGG_PYRIMIDINE_METABOLISM	2.27E-10	1.11E-09
KEGG_COLORECTAL_CANCER	4.38E-10	2.09E-09

KEGG_BASE_EXCISION_REPAIR	9.95E-10	4.63E-09
KEGG_LONG_TERM_POTENTIATION	1.63E-09	7.42E-09
KEGG_PYRUVATE_METABOLISM	1.94E-09	8.57E-09
KEGG_VEGF_SIGNALING_PATHWAY	2.51E-09	1.09E-08
**KEGG_TIGHT_JUNCTION**	2.77E-09	1.17E-08
KEGG_AMINOACYL_TRNA_BIOSYNTHESIS	3.26E-09	1.35E-08
KEGG_JAK_STAT_SIGNALING_PATHWAY	6.37E-09	2.58E-08
KEGG_ACUTE_MYELOID_LEUKEMIA	6.94E-09	2.75E-08
KEGG_B_CELL_RECEPTOR_SIGNALING_PATHWAY	8.92E-09	3.46E-08
KEGG_MELANOMA	1.19E-08	4.39E-08
**KEGG_TGF_BETA_SIGNALING_PATHWAY**	1.19E-08	4.39E-08

Supplementary Table 2

Patient	GSE7599 GATA6	GSE7599 SMAD4	
GSM183763	AMPLIFICATION	LOSS	
GSM183779	GAIN	GAIN	
GSM183764			
GSM183780		LOSS	
GSM183765			
GSM183781		LOSS	
GSM183766	LOSS		ind
GSM183782			
GSM183767			
GSM183783	GAIN	GAIN	
GSM183768			
GSM183784	GAIN	GAIN	
GSM183769	LOSS	GAIN	ind
GSM183785	GAIN	GAIN	
GSM183770		GAIN	
GSM183786	LOSS	GAIN	ind
GSM183778			
GSM183792			
GSM183771	GAIN	LOSS	
GSM183787	LOSS	LOSS	Chr18 loss
GSM183772	GAIN	LOSS	
GSM183788	AMPLIFICATION	LOSS	
GSM183773			
GSM183789	LOSS	LOSS	Chr18 loss
GSM183774			
GSM183790	GAIN	LOSS	
GSM183775			
GSM183791	LOSS	LOSS	ind
GSM183776	AMPLIFICATION	LOSS	
GSM183777		GAIN	

Legend (cell colour): red = LOSS; light blue = GAIN; dark blue = AMPLIFICATION

Patient	GSE25273 GATA6	GSE25273 SMAD4	
GSM621863			
GSM621873		LOSS	
GSM621878	AMPLIFICATION	LOSS	
GSM621883			
GSM621888	LOSS	LOSS	1

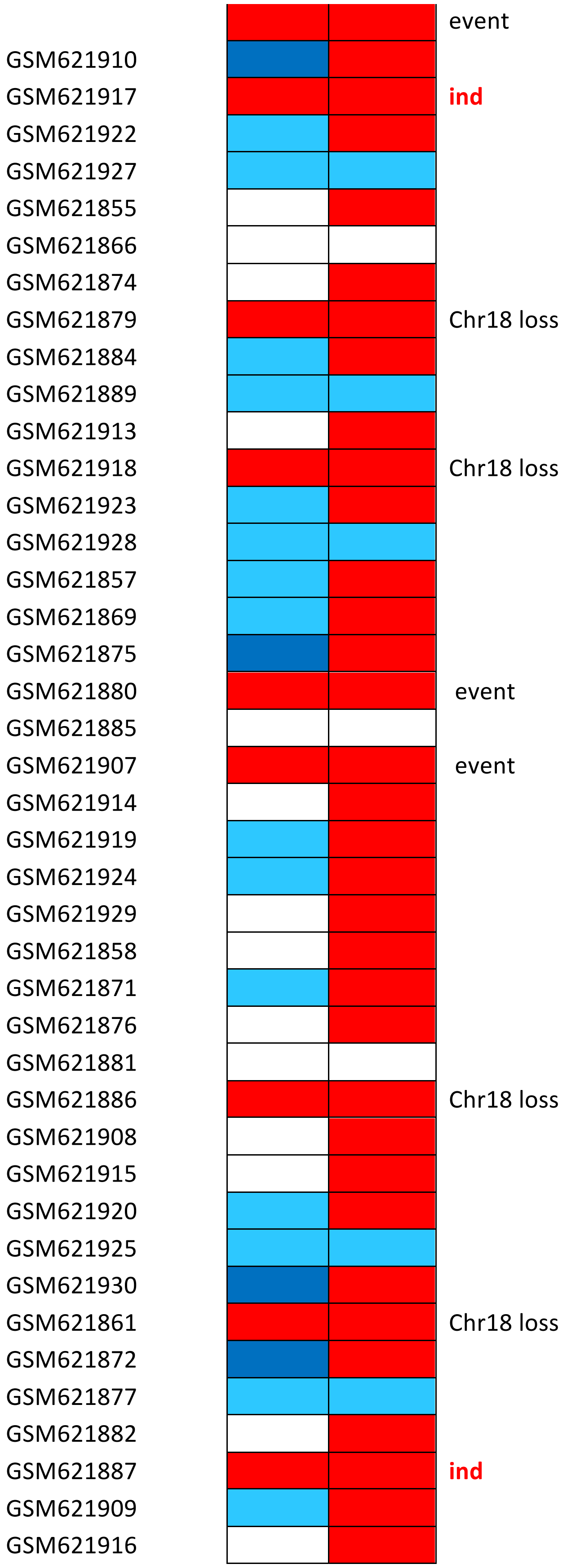
event
GSM621910
GSM621917 ind
GSM621922
GSM621927
GSM621855
GSM621866
GSM621874
GSM621879 Chr18 loss
GSM621884
GSM621889
GSM621913
GSM621918 Chr18 loss
GSM621923
GSM621928
GSM621857
GSM621869
GSM621875
GSM621880 event
GSM621885
GSM621907 event
GSM621914
GSM621919
GSM621924
GSM621929
GSM621858
GSM621871
GSM621876
GSM621881
GSM621886 Chr18 loss
GSM621908
GSM621915
GSM621920
GSM621925
GSM621930
GSM621861 Chr18 loss
GSM621872
GSM621877
GSM621882
GSM621887 ind
GSM621909
GSM621916

E-MTAB-570

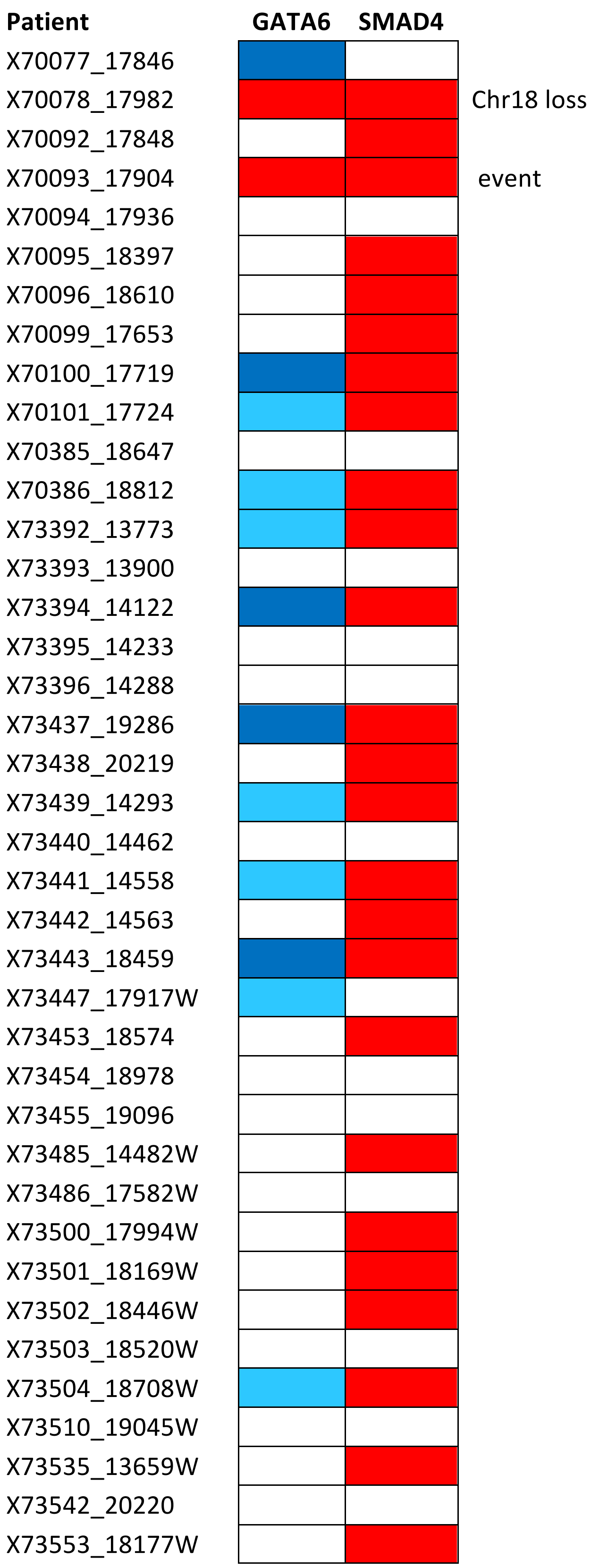
Patient
GATA6
SMAD4
X70077_17846
X70078_17982
X70092_17848
X70093_17904
X70094_17936
X70095_18397
X70096_18610
X70099_17653
X70100_17719
X70101_17724
X70385_18647
X70386_18812
X73392_13773
X73393_13900
X73394_14122
X73395_14233
X73396_14288
X73437_19286
X73438_20219
X73439_14293
X73440_14462
X73441_14558
X73442_14563
X73443_18459
X73447_17917W
X73453_18574
X73454_18978
X73455_19096
X73485_14482W
X73486_17582W
X73500_17994W
X73501_18169W
X73502_18446W
X73503_18520W
X73504_18708W
X73510_19045W
X73535_13659W
X73542_20220
X73553_18177W
Chr18 loss
event

Supplementary Table 3

Gene sets enriched among genes: UP IN GATA6^{low} TUMORS	NES	FDR q-val
SCHUETZ_BREAST_CANCER_DUCTAL_INVASIVE_UP	4.39354	0
**CHARAFE_BREAST_CANCER_LUMINAL_VS_MESENCHYMAL_DN**	4.085699	0
REN_ALVEOLAR_RHABDOMYOSARCOMA_DN	3.33621	0
LINDGREN_BLADDER_CANCER_CLUSTER_2B	3.335549	0
WONG_ADULT_TISSUE_STEM_MODULE	3.264754	0
BOQUEST_STEM_CELL_UP	3.117363	0
NABA_MATRISOME	3.032401	0
LIM_MAMMARY_STEM_CELL_UP	2.955634	1.88E-04
BROWNE_HCMV_INFECTION_48HR_DN	2.949659	1.67E-04
GRUETZMANN_PANCREATIC_CANCER_UP	2.762412	3.20E-04
**CHARAFE_BREAST_CANCER_LUMINAL_VS_BASAL_DN**	2.750357	2.91E-04
**SMID_BREAST_CANCER_LUMINAL_B_DN**	2.574913	6.84E-04
VERHAAK_GLIOBLASTOMA_MESENCHYMAL	2.510614	9.20E-04
LEI_MYB_TARGETS	2.502392	0.00108
MILI_PSEUDOPODIA_HAPTOTAXIS_DN	2.440912	0.00192
DACOSTA_UV_RESPONSE_VIA_ERCC3_DN	2.378595	0.002241
BRUINS_UVC_RESPONSE_LATE	2.32499	0.003478
**SMID_BREAST_CANCER_BASAL_UP**	2.219426	0.007682
KRIEG_HYPOXIA_NOT_VIA_KDM3A	2.18173	0.009221

Supplementary Table 4

	ALL PATIENTS		GATA6 (three categories)						
			LOW		MEDIUM		HIGH		
**Characteristics**	N	(%)	N	(%)	N	(%)	N	(%)	p-value*
**Number**	59	100.0	12	20.3	21	35.6	26	44.1	
**Gender**									0.884
Male	37	62.7	7	58.3	14	66.7	16	61.5	
Female	22	37.3	5	41.7	7	33.3	10	38.5	
**Age at diagnosis**									0.628
Mean (SD)	59	65,1 (10,7)	12	62,9 (10,6)	21	64,6 (11,3)	26	66,5 (10,6)	
Min - Max		42 - 87		45 - 84		45 - 84		42 - 87	
**Tumor site**									0.227
Head&Isthmus	36	61.0	7	58.3	16	76.2	13	50.0	
Body	6	10.2	0	0.0	2	9.5	4	15.4	
Tail	17	28.8	5	41.7	3	14.3	9	34.6	
**Histology**									
**ADK**									0.203
Positive	58	98.3	11	91.7	21	100.0	26	100.0	
Negative	1	1.7	1	8.3	0	0.0	0	0.0	
**Others malignant**									0.075
Positive	4	8.3	1	11.1	3	18.8	0	0.0	
Negative	44	91.7	8	88.9	13	81.2	23	100.0	
missings	*11*	*18.6*	*3*	*25.0*	*5*	*23.8*	*3*	*11.5*	
**Grade of ADK (only for ADK==yes)**									0.394
Well	8	19.5	1	16.7	4	33.3	3	13.0	
Intermediate	18	43.9	4	66.7	3	25.0	11	47.8	
Poor	15	36.6	1	16.7	5	41.7	9	39.1	
missings	*13*	*24.1*	*4*	*40.0*	*6*	*33.3*	*3*	*11.5*	
**Tumor resected**									0.096
Yes	32	54.2	4	33.3	10	47.6	18	69.2	
No	27	45.8	8	66.7	11	52.4	8	30.8	
**Size**$^{\$}$									0.506
Mean (SD)	31	3,1 (0,9)	4	3,5 (0,7)	9	3,2 (0,8)	18	2,9 (1,0)	
Min - Max		14 - 45		3 - 4,5		2,0 - 4,5		0,5 - 5	
missings	*1*		*0*		*1*		*0*		
**T Stage**$^{\$}$									0.184
T1	0	0.0	0	0.0	0	0.0	0	0.0	
T2	0	0.0	0	0.0	0	0.0	0	0.0	
T3	30	93.8	4	100.0	8	80.0	18	100.0	
T4	2	6.2	0	0.0	2	20.0	0	0.0	
**N Stage**$^{\$}$									0.443

Positive	26	81.2	4	100.0	9	90.0	13	72.2	
Negative	6	18.8	0	0.0	1	10.0	5	27.8	
**Chemotherapy**									
**Gemzar**									0.118
Yes	26	44.8	3	25.0	8	38.1	15	60.0	
No	32	55.2	9	75.0	13	61.9	10	40.0	
missing	1	1.7	0	0.0	0	0.0	1	3.8	
**Folfirinox**									0.112
Yes	20	34.5	6	5.0	12	57.1	20	80.0	
No	38	65.5	6	50.0	9	42.9	5	20.0	
missing	1	1.7	0	0.0	0	0.0	1	3.8	
**Other**									1.000
Yes	9	15.5	2	16.7	3	14.3	4	16.0	
No	49	84.5	10	83.3	18	85.7	21	84.0	
missing	1	1.7	0	0.0	0	0.0	1	3.8	

** p-value, Fisher's test for qualitative variables. One-way ANOVA test for age and tumor size.*

[$] Data available o*nly for resected patients N=32*

Supplementary Table 5

		ALL PATIENTS	GATA6 score median (IQR)	GATA6 LOW	GATA6 MEDIUM	GATA6 HIGH	*P* value
**Resection Margin**	Negative	169	205 (157-260)	19	60	90	0.353
	Positive	144	188 (143-253)	18	61	65	
**Lymph Node Status**	Negative	61	223 (157-265)	6	19	36	0.254
	Positive	252	190 (148-250)	31	102	119	
**Stage**	1	17	223 (140-260)	2	5	10	0.338
	2	76	213 (171-275)	7	24	45	
	3	209	190 (145-245)	24	90	95	
	4	8	217 (113-265)	2	2	4	
**Tumour Grade**	Well	21	185 (155-250)	3	8	10	**0.005**
	Moderate	206	210 (160-260)	16	74	116	
	Poor	79	180 (103-227)	16	36	27	
**Local Invasion**	No	165	205 (157-257)	18	59	88	0.422
	Yes	146	190 (145-253)	18	61	67	
**Maximum Tumour diameter**	<30mm	135	190 (148-260)	13	57	65	0.481
	≥30mm	160	196 (148-253)	22	60	78	
**Diabetes**	No	238	192 (150-255)	25	97	116	0.329
	IDDM	66	203 (143-260)	10	21	35	
**Gender**	Male	178	196 (148-255)	23	69	86	0.761
	Female	125	200 (150-258)	14	52	69	
**Age (Years)**	<64	156	199 (144-260)	22	56	78	0.368
	≥64	157	197 (160-252)	15	65	**77**	

Supplementary Table 6

Characteristic		Chemotherapy 5-Fluorouracil/ folinic acid N=150	Chemotherapy Gemcitabine N=163	Total N=313
Age Median (IQR) years		62 (55-70)	64 (56-70)	64 (57-70)
Sex	Female	66 (44%)	69 (42%)	135 (43%)
	Male	84 (56%)	94 (58%)	178 (57%)
Baseline Performance Score	0	57 (38%)	55 (34%)	112 (36%)
	1	79 (53%)	89 (55%)	168 (54%)
	2	14 (9%)	19 (12%)	33 (11%)
Diabetic	No	114 (79%)	124 (78%)	238 (78%)
	NIDDM	11 (8%)	13 (8%)	24 (8%)
	IDDM	19 (13%)	23 (14%)	42 (14%)
Smoking status	Never	60 (43%)	63 (43%)	123 (43%)
	Past	52 (37%)	60 (41%)	112 (39%)
	Present	27 (19%)	25 (17%)	52 (18%)
Post-operative complications	No	114 (77%)	126 (78%)	240 (77%)
	Yes	34 (23%)	36 (22%)	70 (23%)
Hospital stay	Number	139	153	292
Median (IQR) days		13 (10-18)	13 (10-17)	13 (10-17)
Post-Op. CA 19-9	Number	114	117	231
Median (IQR) KU/l		34 (11-81)	25 (11-60)	28 (11-71)
Surgery to Randomisation Median (IQR) days		49 (35-62)	50 (38-61)	49 (37-61)
Surgery	Whipples resection	78 (53%)	82 (51%)	160 (52%)
	Pylorus Preserving	57 (39%)	66 (41%)	123 (40%)

	**Total Pancreatectomy**	6 (4%)	4 (2%)	10 (3%)
	**Distal Panc.**	7 (5%)	10 (6%)	17 (5%)
**Extent of resection**	**Standard**	116 (81%)	110 (69%)	226 (75%)
	**Radical resection**	17 (12%)	27 (17%)	44 (15%)
	**Extended radical**	11 (8%)	22 (14%)	33 (11%)
**Maximum tumour diameter, mm Median (IQR)**		27 (22-35)	30 (22-40)	30 (22-37)
**Tumour grade**	**Well**	8 (5%)	13 (8%)	21 (7%)
	**Moderately**	98 (67%)	108 (68%)	206 (67%)
	**Poorly**	41 (28%)	38 (24%)	79 (26%)
**Lymph node invasion**	**Negative**	30 (20%)	31 (19%)	61 (19%)
	**Positive**	120 (80%)	132 (81%)	252 (81%)
**Resection margins**	**Negative**	74 (49%)	95 (58%)	269 (54%)
	**Positive**	76 (51%)	68 (42%)	144 (46%)
**Local Invasion**	**No**	83 (56%)	85 (52%)	1688 (54%)
	**Yes**	65 (44%)	77 (48%)	142 (46%)
**Tumour stage**	**I**	10 (7%)	7 (4%)	17 (5%)
	**II**	35 (23%)	41 (25%)	76 (25%)
	**III**	97 (65%)	112 (70%)	209 (67%)
	**IVa**	7 (5%)	1 (1%)	8 (3%)

Supplementary Table 7

		Hazard Ratio (95% Confidence Interval)		
		Chemotherapy		Total
**Characteristic**		**5-fluorouracil /folinic acid**	**Gemcitabine**	
Resection Margin		**N=150**	**N=163**	**N=313**
	Negative	1	1	1
	Positive	1.54 (1.27-2.66)	1.26 (0.90-1.78)	1.52 (1.18-1.94),
		Wald χ^2= 10.38, *P*=**0.001**	Wald χ^2= 1.78, *P*=0.182	Wald χ^2= 10.76, *P*=**0.001**
Lymph Node Status		**N=150**	**N=163**	**N=313**
	Negative	1	1	1
	Positive	2.41 (1.45-3.99)	1.73 (1.08-2.75)	2.05 (1.45-2.89)
		Wald χ^2= 11.55, *P*=**0.001**	Wald χ^2= 5.27, *P*=**0.022**	Wald χ^2= 16.75, *P*<**0.001**
Stage		N=149	N=161	N=310
	2-Jan	1	1	1
	4-Mar	1.50 (1.00-2.25)	1.45 (0.99-2.12)	1.48 (1.12-1.95)
		Wald χ^2= 3.91**,** ***P*=0.048**	Wald χ^2= 3.63, *P*=0.057	Wald χ^2= 7.57, *P*=**0.006**
Tumour Grade		N=147	N=159	N=306
	Well	1	1	1
	Moderately	0.98 (0.43-2.24)	1.05 (0.56-1.96)	1.03 (0.63-1.70)
	Poorly	1.05 (0.43-2.52)	1.51 (0.76-2.99)	1.27 (0.74-2.17)
		Wald χ^2= 0.10, *P*=0.951	Wald χ^2= 3.34, *P*=0.188	Wald χ^2= 2.13, *P*=0.344
Local Invasion		N=149	N=162	N=311

	No	1	1	1
	Yes	1.79 (1.24-2.59)	1.30 (0.92-1.83)	1.52 (1.19-1.95)
		Wald χ^2= 9.51, ***P*=0.002**	Wald χ^2= 2.27, *P*=0.132	Wald χ^2= 10.86, ***P*=0.001**
Maximum Tumour diameter		N=142	N=153	N=295
	<30mm	1	1	1
	≥30mm	1.35 (0.93-1.96)	1.38 (0.96-1.98)	1.36 (1.05-1.76)
		Wald χ^2= 2.55, *P*=0.111	Wald χ^2= 3.08, *P*=0.079	Wald χ^2= 5.51, ***P*=0.019**
Diabetes mellitus		N=144	N=160	N=304
	No	1	1	1
	Yes	1.02 (0.65-1.61)	1.03 (0.68-1.57)	1.03 (0.75-1.40)
		Wald χ^2= 0.01, *P*=0.933	Wald χ^2= 0.02, *P*=0.879	Wald χ^2= 0.03, *P*=0.870
Gender		N=150	N=163	N=313
	Male	1	1	1
	Female	1.18 (0.82-1.69)	1.09 (0.78-1.54)	1.13 (0.88-1.45)
		Wald χ^2= 0.76, *P*=0.383	Wald χ^2= 0.25, *P*=0.617	Wald χ^2= 0.98, *P*=0.323
Age, years		N=150	N=163	N=313
	≥64	1	1	1
	<64	1.48 (1.03-2.14)	1.01 (0.72-1.42)	1.22 (0.95-1.56)
		Wald χ^2= 4.38**,** ***P*=0.036**	Wald χ^2= 0.01, *P*=0.942	Wald χ^2= 2.40, *P*=0.121
Smoking		N=139	N=148	N=287
	Never	1	1	1
	Ex	1.06 (0.70-1.61)	1.29 (0.87-1.92)	1.17 (0.88-1.56)

	Current	1.04 (0.63-1.71)	1.45 (0.88-2.39)	1.21 (0.85-1.73)
		Wald χ^2= 0.07, *P*=0.964	Wald χ^2= 2.68, *P*=0.261	Wald χ^2= 1.68, *P*=0.432
GATA6		N=150	N=163	N=313
	High	1	1	1
	Medium	1.49 (1.01-2.20)	0.97 (0.67-1.39)	1.19 (0.91-1.55)
	Low	1.73 (0.99-3.03)	0.99 (0.56-1.72)	1.27 (0.86-1.89)
		Wald χ^2= 5.72, *P*=0.057	Wald χ^2= 0.04, *P*=0.982	Wald χ^2= 2.38, *P*=0.304
KRT14		N=139	N=139	N=278
	neg	1	1	1
	pos	1.09 (0.71-1.67)	1.11 (0.70-1.74)	1.10 (0.81-1.49)
		Wald χ^2= 0.15, *P*=0.701	Wald χ^2= 0.19, *P*=0.660	Wald χ^2= 0.34, *P*=0.558

Supplementary Table 8

		5-fluorouracil /folinic acid (n=139)			Gemcitabine (n=139)		
**Variable**		**HR (95% CI)**	**Wald χ^2**	**p**	**HR (95% CI)**	**Wald χ^2**	**P**
Resection Margin	**Negative**	1	6.23	**0.013**	1	0.97	0.326
	**Positive**	1.64 (1.11-2.41)			1.19 (0.84-1.69)		
Lymph Node Status	**Negative**	1	6.45	**0.011**	1	1.96	0.162
	**Positive**	2.35 (1.22-4.53)			1.55 (0.84-2.88)		
Stage	**1/2**	1	0.26	0.609	1	0.12	0.728
	**3/4**	0.87 (0.51-1.48)			1.09 (0.66-1.81)		
Local Invasion	**No**	1	4.5	**0.032**			
	**Yes**	1.50 (1.04-2.17)					
Age	**>=64**	1	4.87	**0.027**			
	**<64**	1.55 (1.05-2.29)					
GATA6_CLASS	**High**	1	1.77	0.413	1	0.02	0.992
	**Medium**	1.30 (0.87-1.95)			0.97 (0.54-1.73)		
	**Low**	1.25 (0.67-2.32)			0.98 (0.68-1.42)		

Supplementary Table 9

	GATA6	***KRAS***	***CDKN2A***	***TP53***	***SMAD4***
**A13B**	Amplified	MUT	*HD*	HD	WT
**PaTu8988S**	WT	MUT	*HD*	MUT	HD
**SK-PC-1**	WT	MUT	*HD*	MUT	HD
**L3.6pl**	WT	MUT	*Meth*	WT	HD
**Panc1** (used in ref. 17)	WT	MUT	*HD*	MUT	WT

ref:
Schuhmacher et al. Clin. Can. Res. 1999
Hamidi et al. Br. J. Cancer 2014
Moore et al. Virchows Arch. 2001
Jones et al. Science 2008

Supplementary Table 10

Target protein	Provider	Cat. Number	Use
GATA6	R&D	AF1700	1:1000 WB
			1:1000 IHC
			1 μg/ChIP
	Santa Cruz	sc-9055	1:200 IF
E-cadherin	BD Laboratories	610182	1:1000 WB
			1:1000 IHC
			1:100 IF
vimentin	Millipore	AB5733	1:100 IF
	Santa Cruz	sc-6260	1:1000 WB
FOXA1	Abcam	ab-23738	1:500 WB
FOXA2	Seven Hills	WRAB-1200	1:2000 IHC
	Santa Cruz	sc-6554	1:500 WB
			1 μg/ChIP
GAPDH	Home made		1:1000 WB
Vinculin	SIGMA	V9131	1:3000 WB
KRT5	Covance	PRB-16P	1:500 WB
KRT14	Covance	PRB-155P	1:500 WB
			1:1000 IHC

Supplementary Table 11

RT-qPCR

hCDH1_F	AGAACGCATTGCCACATACACTC
hCDH1_R	CATTCTGATCGGTTACCGTGATC
hCLDN1_ex2-3_F	GTGCTTGGAAGACGATGAGG
hCLDN1_ex2-3_R	CATACCATGCTGTGGCAACT
hCRB3_F	TTCTGCAAATGAGAATAGCACTG
hCRB3_R	GAGGGAGAAGACCACGATGA
hCTNNB1_ex1-2_F	GTCGAGGACGGTCGGACT
hCTNNB1_ex1-2_R	CCATTGTCCACGCTGGATT
hEGFR__ex24-25_F	CCCAAAGTTCCGTGAGTTGA
hEGFR__ex24-25_R	TCGTCCATGTCTTCTTCATCC
hFOXA1_F	GCTCCAGGATGTTAGGAACTGT
hFOXA1_R	ATGGTCATGTAGGTGTTCATGG
hFOXA2_F	AGCTACTATGCAGAGCCCGA
hFOXA2_R	TACGTGTTCATGCCGTTCAT
hGATA6_F	GTGCCCAGACCACTTGCTAT
hGATA6_R	TGGAATTATTGCTATTACCAGAGC
hHNF4a__F	GGCTGCAAGGGCTTCTTC
hHNF4a__R	GGCACTGGTTCCTCTTGTCT
hHPRT_F	GGCCAGACTTTGTTGGATTTG
hHPRT_R	TGCGCTCATCTTAGGCTTTGT
hKRas_ex1-2_F	TGTGGTAGTTGGAGCTGGTG
hKRas_ex1-2_R	TCGAGAATATCCAAGAGACAGG
hMAP2K2_ex1-2_F	GCTCACCATCAACCCTACCA
hMAP2K2_ex1-2_R	GTCAAGTTCCAGCTCCTCCA
hNFKB1_ex2-3	TGTTTCATTTGGATCCTTCTTTG
hNFKB1_ex2-3	ATTTGAAGGTATGGGCCATCT
hRELA_ex1-3_F	GCGAATGGCTCGTCTGTAGT
hRELA_ex1-3_R	TCCACATAGGGGCCAGAG
hSNAI1_ex1-2_F	GCGAGCTGCAGGACTCTAAT
hSNAI1_ex1-2_R	CGGTGGGGTTGAGGATCT
hSNAI1_F	CAAGATGCACATCCGAAGC
hSNAI1_R	CAGTGGGGACAGGAGAAGG
hSOX9_ex2-3_F	ACTCGCCACACTCCTCCTC
hSOX9_ex2-3_R	GTCGGTTTTGGGGGTGGT
hTWIST1_F	ACCCAGTCGCTGAACGAG
hTWIST1_R	TGGAGGACCTGGTAGAGGAA
hVIM_F	TCAGAGAGAGGAAGCCGAAA
hVIM_R	CAAAGATTCCACTTTGCGTTC
hZO1_ex3-4_F	CAGAGTGGGGAAACGTCAAT
hZO1_ex3-4_R	CACTTTTCCTTAGTTGCTGAACA

ChIP-qPCR

hCDH1 +560bp_F	AGAGGGGCATCCGTAGAAAT
hCDH1 +560bp_R	TCTCCAGTACCCCACTTTCC
hCLDN1_prom_F	AAACCAGGATTCTCTTCATTTCC
hCLDN1_prom_R	TCCCTTATGGGCTCATTCTG
hCRB3_prom2_F	CCTGGGGGCAAAGTAGGTA
hCRB3_prom2_R	CCAGATACCCAGCGCTTCTA
hCTNNB1TSS_F	GCGCCATTTTAAGCCTCTC
hCTNNB1TSS_R	CTGAAGCTGCTCCTCAGACC
hECAD_TSS_F	GTGAACCCTCAGCCAATCAG
hECAD_TSS_R	TCACAGGTGCTTTGCAGTTC
hEGFR_-400_F	AGAGGGTCCCGTAGTGCTG
hEGFR_-400_R	AGACTGGCCGAGCCTTAGAG
hGATA6 TSS_F	TACTGCTCTGCCGGAAAACT
hGATA6 TSS_R	GCAGTTCCGACCCACAGC
hHNF4a_TSS_F	TGGGTGATTAGAAGAATCAATAAGA
hHNF4a_TSS_R	GCTCACAGCAGCAGCACA
hKRAS_TSS2_F	CCTCAGGGTCGGCCTATAC
hKRAS_TSS2_R	GTCTCCCTAAGTCCCCGAAG
hMAP2K2_TSS_F	CCTCGCTTGCCTTACCAC
hMAP2K2_TSS_R	ACCTGTGCACGACCATCAC
hNFKB1_TSS_F	TGGACCGCATGACTCTATCA
hNFKB1_TSS_R	CTGGAGCCGGTAGGGAAG
hNOTCH1TSS_1_F	GGCGGGGAGAAATGTAAGAG
hNOTCH1TSS_1_R	GAGGGGTTGCGTAAGAAGC
hRELA_prom_F	CGGATGGGACGACTGAGG
hRELA_prom_R	ATGAGAGCCGGCAGTTTG
hSNAI1_TSS_F	GGAGACGAGCCTCCGATT
hSNAI1_TSS_R	GCCGCCAACTCCCTTAAGTA
hSOX9_intr2_F	CACAAGTAGCAATTAGGTCTTCC
hSOX9_intr2_R	GGAGATAAGTCGCCCAATGA
hVIM_prom_F	AGCCCGCTGAGACTTGAAT
hVIM_prom_R	GGTGGGGTCGCTTAGTCAC
hZEB1_prom_F	AGGCGTGGGACTGATGGTAG
hZEB1_prom_R	ATTCTCCCTGTACCCTGTGC
hZO1_prom_F	AAGAAAACCCGACCTACTACGC
hZO1_prom_R	TGCTGTCTTTGGAGGAGTGG
NEG_F	CATTGGGAAGTGATGATGTGATCT
NEG_R	GTCCTCTCTGCCATCTTCACTCA

SUPPLEMENTARY FIGURES

Supplementary Figure 1. GATA6 expression in PDAC cells. (A) RT-qPCR analysis of GATA6 mRNA levels in a panel of PDAC cell lines, relative to HPRT. In red are the cell lines that were used in subsequent experiments. (B) Western blot showing the protein levels of GATA6, E-cadherin, and vimentin. Vinculin was used as loading control.

Supplementary Figure 1

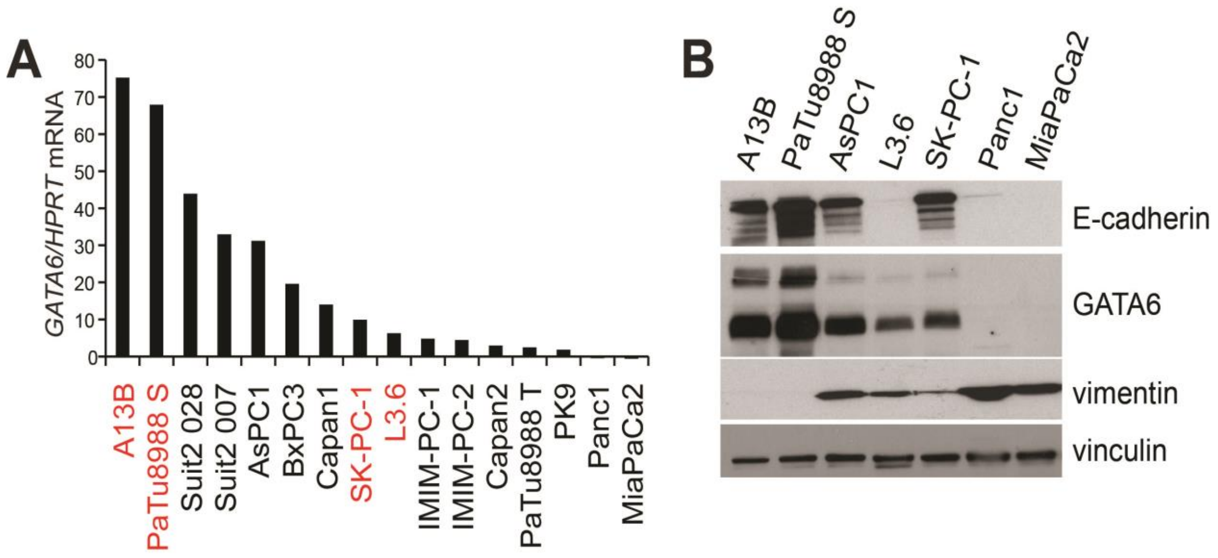

Supplementary Figure 2. GATA6 knock-down and overexpression. (A-C) Top: microphotographs showing the PDAC cell lines that were used for *GATA6* knock-down experiments. Bottom: GATA6 expression in A13B, PaTu8988S, SK-PC-1 infected with the indicated constructs, measured by RT-qPCR. (D) Expression of GATA6, E-cadherin, and vimentin detected by western blotting in total lysates from A13B and SK-PC-1 cells infected with the indicated constructs. GAPDH was used as a loading control. (E) Quantification of the *in vitro* invasiveness of SK-PC-1 cells infected with the indicated constructs. (F) Microphotograph of parental L3.6pl cells, used for GATA6 overexpression, and (bottom) mRNA levels of GATA6 in L3.6pl cells infected with the indicated constructs. Data are presented as mean±s.e.m. of at least three independent experiments; **P*<0.05, ***P*<0.01.

Supplementary Figure 2

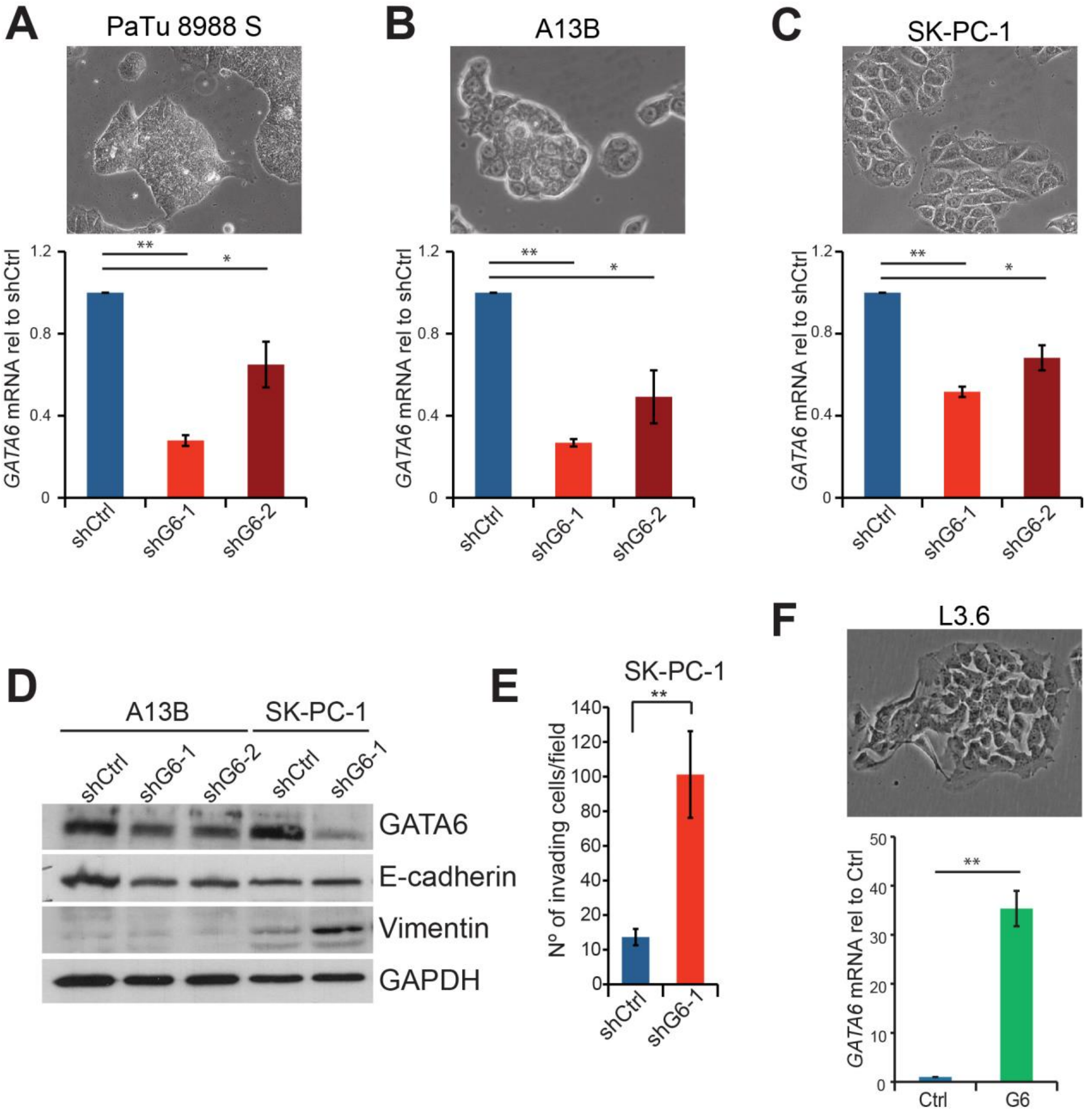

Supplementary Figure 3. GATA6 expression is lower in metastases compared to primary tumors. Boxplot showing the expression of GATA6 in normal pancreas (norm, adjacent to PDAC), primary tumors (PDAC), and metastases (met) in the patients' series described by Moffitt et al. ***P*<0.001

Supplementary Figure 3

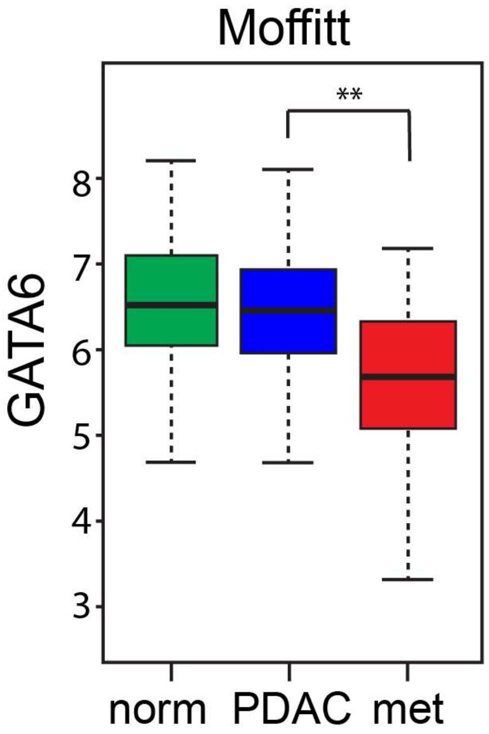

Supplementary Figure 4. GATA6 ChIP-Seq validation and ChIP analysis of FOXA2 on GATA6 targets. (A) Top: Representative genomic distribution of GATA6 in a region of human chromosome 14, as displayed in the UCSC genome browser. Tag counts (top-red for GATA6-ChIP and blue for input) and called peaks (bottom) tracks are shown. Bottom: core matrices obtained for the most significantly enriched motifs found in the GATA6 ChIP-Seq peaks. (B) Distribution of GATA6 ChIP-Seq peaks according to their distance from the closest transcription start site (TSS). (C) GATA6 binding to the promoter region of the indicated genes detected by ChIP-qPCR in PaTu8988S cells, represented as % of input chromatin and compared with non-specific IgG binding. Data are shown as mean±s.e.m of at least three independent experiments; **P*<0.05, ***P*<0.01. (D) Representation of the ChIPSeq peaks found in the promoter of SNAI1, ZEB1, ZEB2, and VIM, as displayed in the UCSC genome browser. Scale bar: 5Mb. (E) FOXA2 binding to the promoter of the indicated genes, selected as GATA6 targets. UP and DOWN refers to the regulation of the corresponding mRNAs in GATA6-silenced PaTu8988S cells. Data are presented as mean±s.e.m. of at least three independent experiments; **P*<0.05, ***P*<0.01.

Supplementary Figure 4

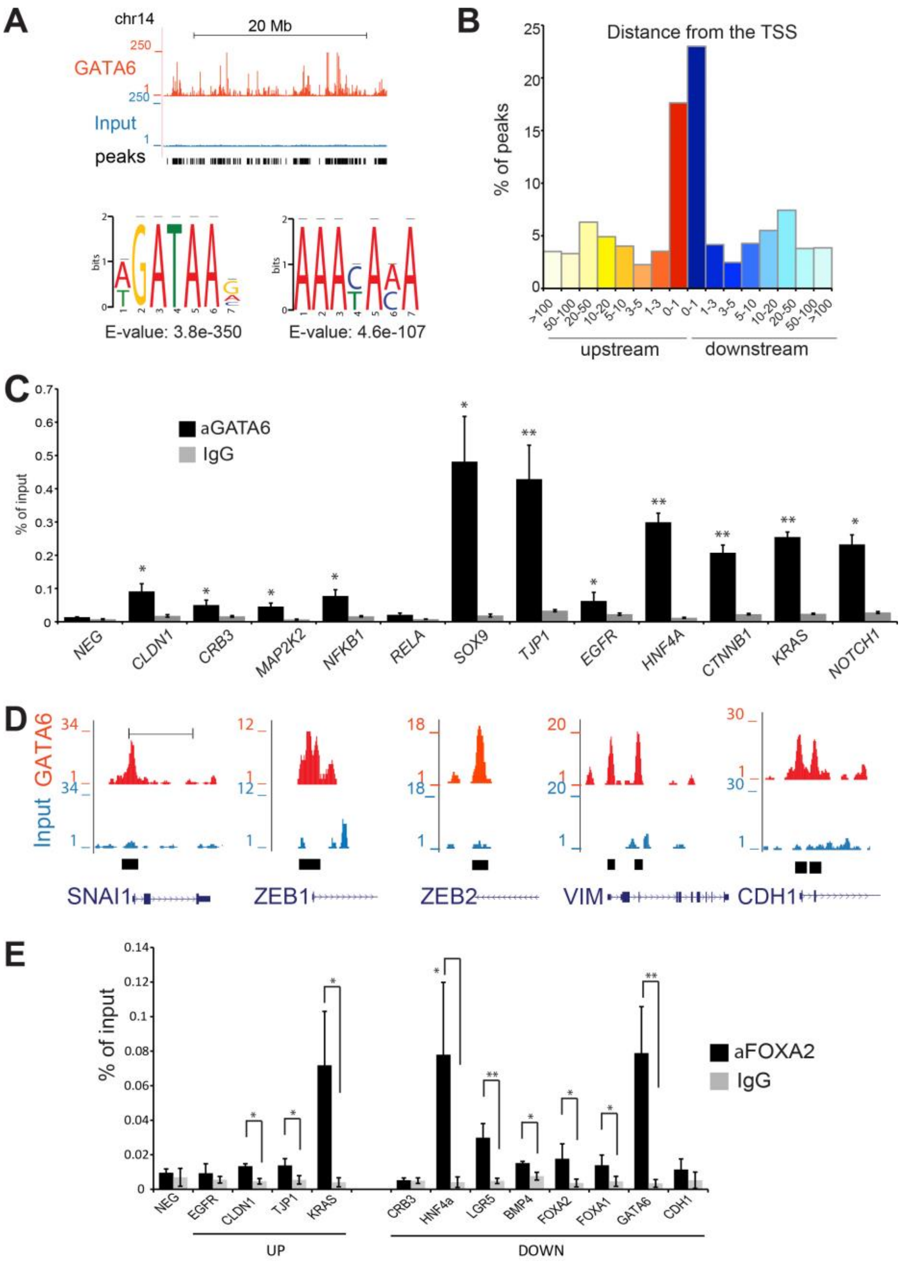

Supplementary Figure 5. GATA6 correlates with the epithelial phenotype in primary tumors. Scatter plots showing correlated expression of GATA6, FOXA2, and E-cadherin mRNA in the Moffitt series. *P*<0.001 for all the comparisons.

Supplementary Figure 5

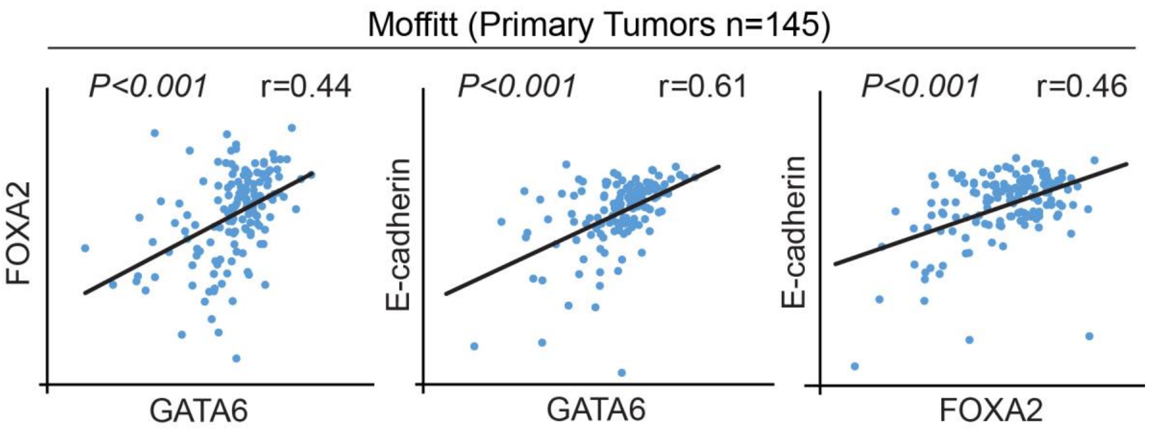

Supplementary Figure 6. $GATA6_{low}$ tumors display upregulation of pathways associated with basal breast cancer. GSEA plots showing some of the genesets that were most enriched within the genes upregulated in $GATA6_{low}$ tumors.

Supplementary Figure 6

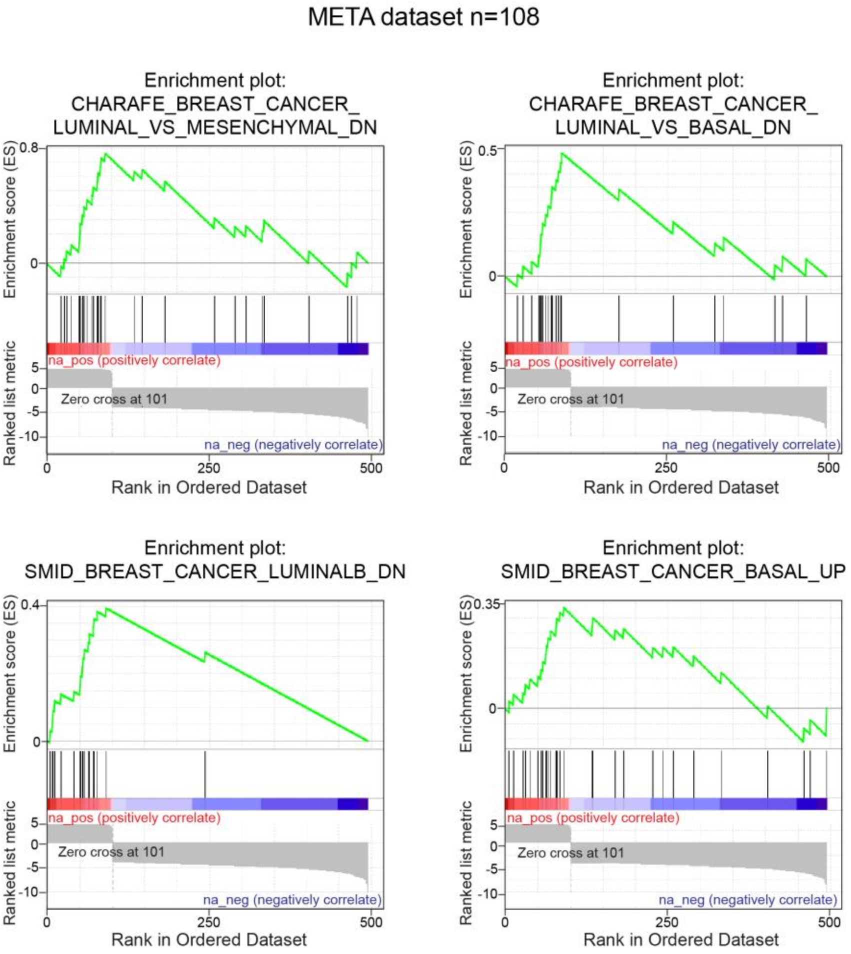

Supplementary Figure 7. Low GATA6 is detected in a BAS-L subtype of PDACs. (A) Heat map showing the expression of the BASE47 signature in a meta-dataset of 108 PDAC samples; clustering was performed with the Hierarchical Clustering tool of the GenePattern suite. (B) Boxplot showing the expression of GATA6 in the two groups classified by the BASE47 signature. ***P*<0.0001.

Supplementary Figure 7

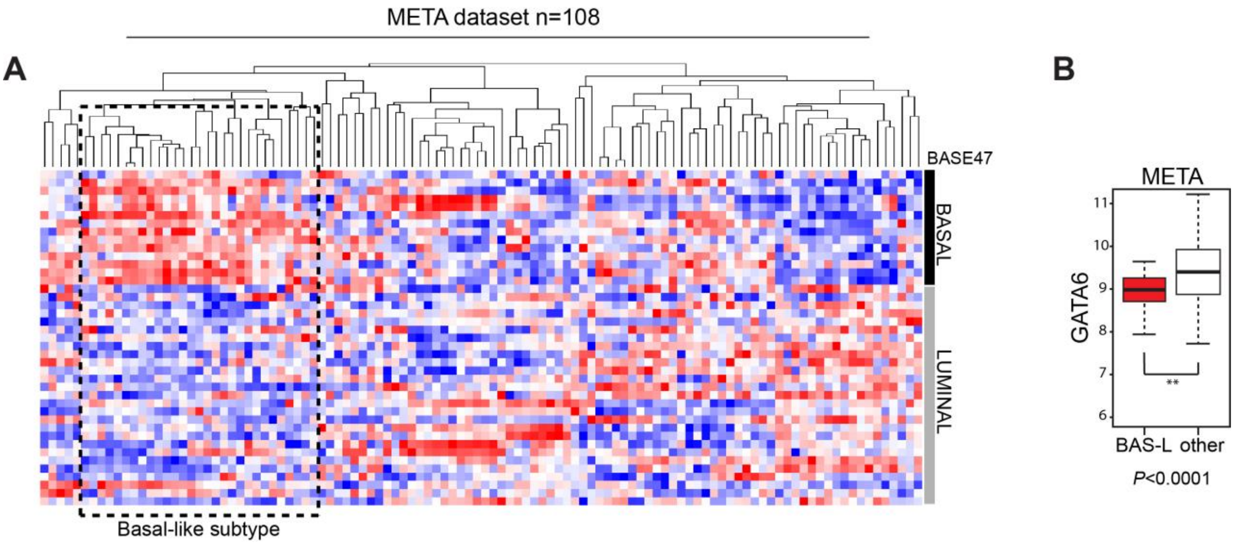

Supplementary Figure 8. GATA6low cells are insensitive to 5-FU. (A) WB showing the expression of GATA6 in the TKCC cells. GAPDH was used as loading control. (B-D) Scatter plots showing cell survival upon treatment with the indicated doses of 5-FU (B), gemcitabine (C), and paclitaxel (D), plotted against GATA6 protein level. Red square indicates the dose/drug showing significant correlation. Survival was normalized against DMSO-treated cells. Data are presented as median value of at least three independent experiments.

Supplementary Figure 8

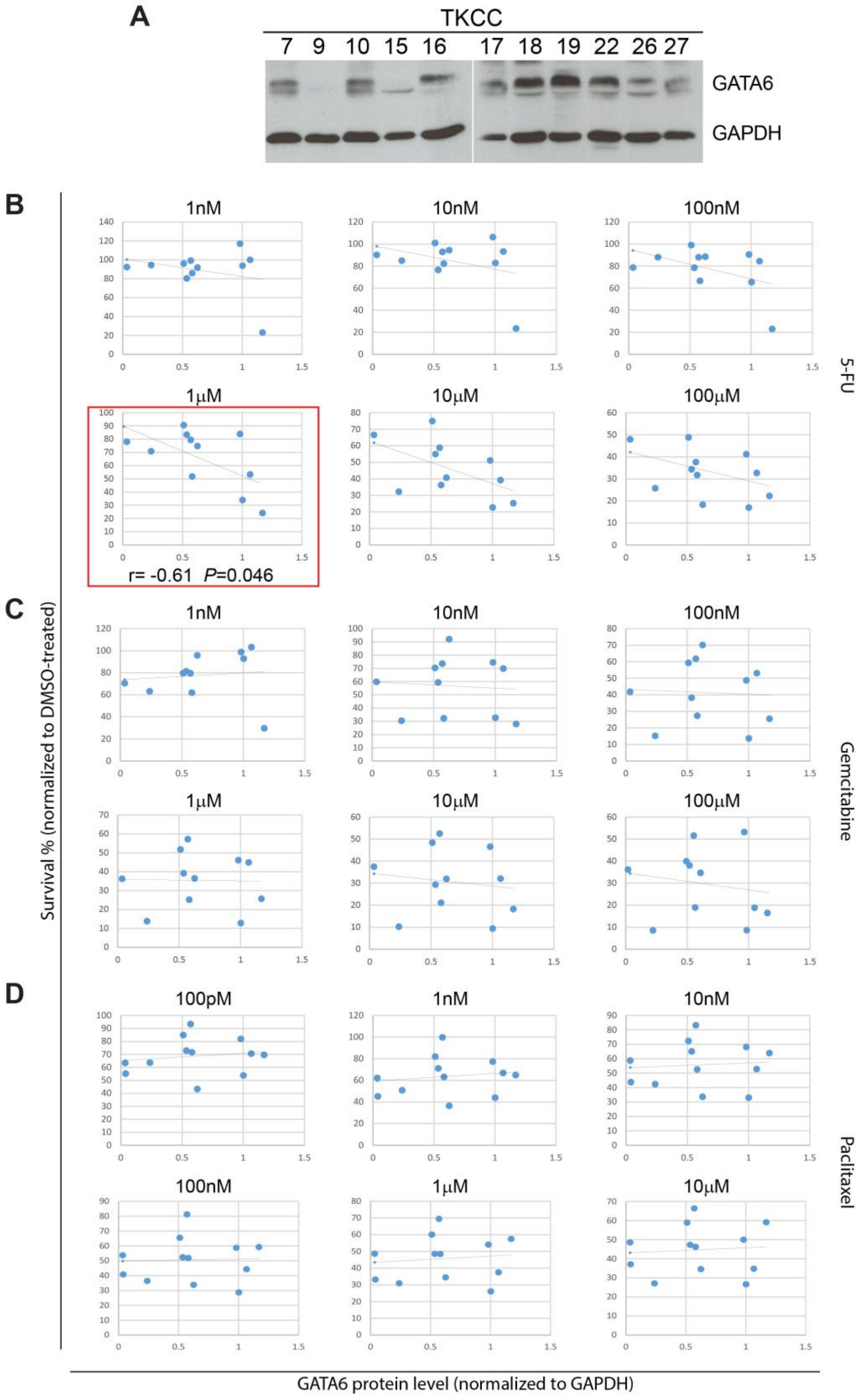

Supplementary Figure 9. GATA6 modulation does not change drug sensitivity of PaTu8988S and L3.6pl cells. (A) Western blot showing the extent of GATA6 overexpression and knock-down. Vinculin was used as loading control. (B) Survival of the indicated cells after treatment with increasing doses of either 5-FU or gemcitabine.

Supplementary Figure 9

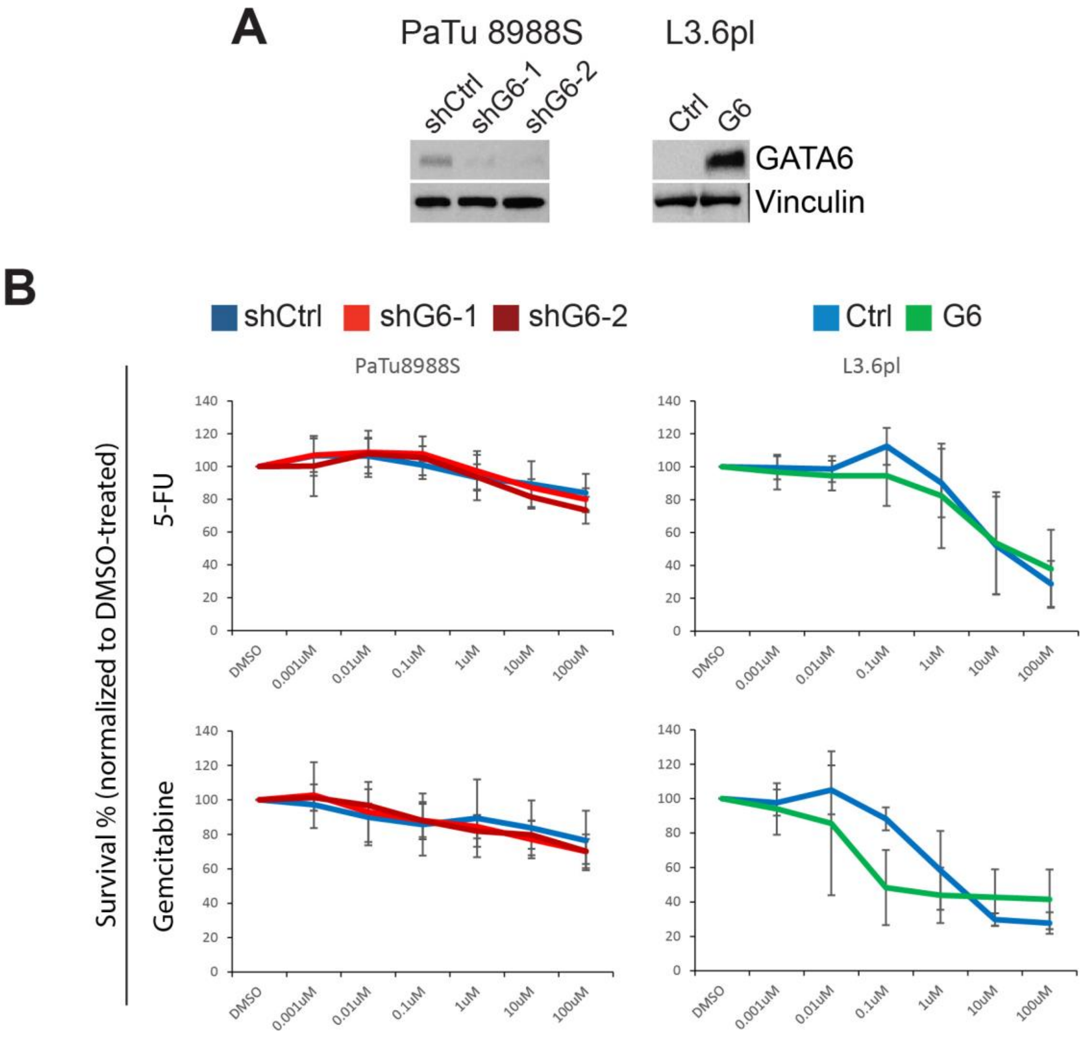

Supplementary Figure 10. GATA6 modulation does not change drug sensitivity of TKCC cells. (A) Western blot showing the extent of GATA6 overexpression and knockdown. GAPDH was used as loading control. (B) Survival of the indicated cells after treatment with increasing doses of either 5-FU or gemcitabine.

Supplementary Figure 10

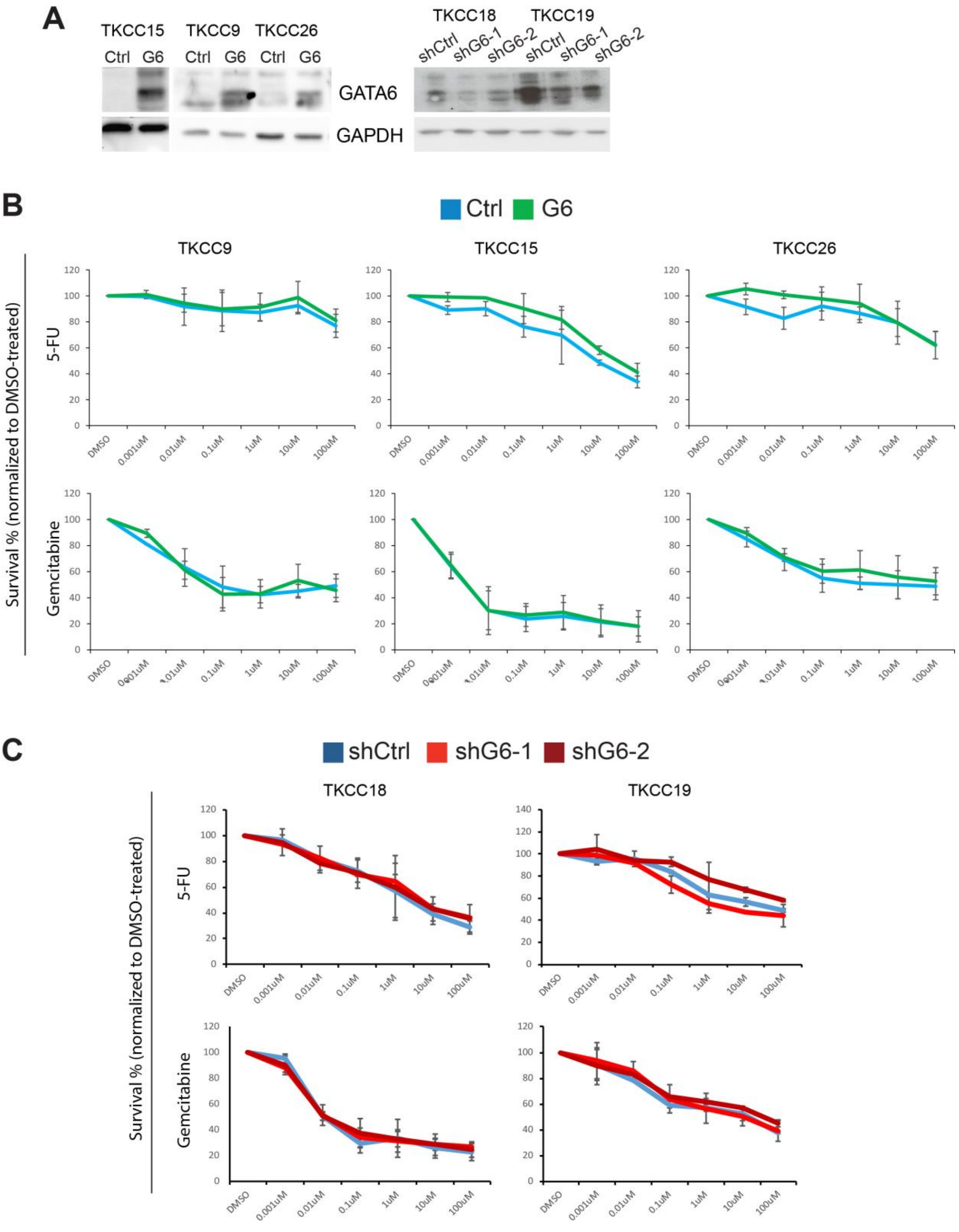

Supplement: Supplementary data [file gutjnl-2015-311256supp001.pdf]
